# From Benchtop to Handheld Systems: Calibration Transfer for On-Site NIR System Butter Quality Monitoring

**DOI:** 10.3390/s26196110

**Published:** 2026-09-26

**Authors:** Lorenzo Serva, Andrea Calore, Severino Segato, Veronica Ferrari, Saowaluk Rungchang, Paolo Berzaghi, Giulio Cozzi

**Affiliations:** 1 Department of Animal Medicine, Production and Health, University of Padova, Viale dell’Università 16, 35020 Legnaro, Italy; lorenzo.serva@unipd.it (L.S.); andrea.calore.5@studenti.unipd.it (A.C.); veronica.ferrari@unipd.it (V.F.); paolo.berzaghi@unipd.it (P.B.); giulio.cozzi@unipd.it (G.C.); 2 Division of Food Science and Technology, Faculty of Agro-Industry, Chiang Mai University, Chiang Mai 50100, Thailand; saowaluk.run@cmu.ac.th

**Keywords:** fatty acid profile, spectroscopy portable instruments, pre-treatments, spectra, transfer algorithms

## Abstract

**Highlights:**

**What are the main findings?**

- DS25 calibrations were transferable to handheld NIR instruments.
- Transfer performance depended on instrument, chemical variable, and transfer method.

**What are the implications of the main findings?**

- Handheld NIR can support rapid on-site butter chemical quality monitoring.
- Each device still requires instrument-specific validation before routine use.

**Abstract:**

Butter quality assessment requires rapid and reliable portable analytical tools that enable on-site analysis beyond specialized food laboratories. This study evaluated the transfer of calibration models developed on a benchtop Near-Infrared (NIR) instrument (DS25) to three handheld NIR devices (Aurora, JDSU and ISC) for the prediction of butter compositional traits and fatty acid profile. Ninety-nine butter samples from different milk production systems were used for spectral acquisition and calibration development. Different calibration transfer (CT) strategies, including spectral bias correction (SBC), direct standardization (DS), piecewise direct standardization (PDS), Shenk–Westerhaus (S-W) standardization and dynamic orthogonal projection (DOP), were combined with Partial Least Squares (PLS) regression and evaluated on a hold-out validation set. Across both hold-out validation and prediction-based consistency analyses, Aurora and JDSU showed the most robust performance relative to the DS25 benchmark and the multi-instrument consensus, particularly for moisture, fat content, and major fatty acids (FA) groups, whereas ISC showed lower and more variable performance. The outcomes suggested that handheld NIR spectroscopy, supported by appropriate calibration transfer and limited target-instrument updating, can be used for rapid and decentralized butter quality monitoring. However, transfer success was instrument-, variable- and method-dependent, confirming the need for device-specific validation before routine application.

## 1. Introduction

Butter is one of the most valuable dairy products, and its chemical and nutritional quality is primarily determined by the amount of fat and its fatty acid profile, which in turn depends on a complex interaction among the cows’ feeding and rearing system and milking season. For instance, butter from mountain grazing-based fed animals has a relevant market value because it is associated with local identity, short supply chains, and more natural and traditional (pasture-based) cattle production systems. Indeed, mountain cheese and dairy products are often perceived as high-quality foods because of their link with extensive farming, animal grazing, pasture botanical diversity, and traditional processing [1]. In the Alpine context, these products also have environmental, social, and cultural value, supporting the maintenance of mountain livestock systems and contributing to the economic sustainability of marginal rural areas [2,3]. The official European “mountain product” indication further emphasizes the importance of geographical origin and production context in generating added value for dairy products [3]. Butter quality is strongly influenced by the milk fatty acids (FA) composition, which has been demonstrated to serve as a reliable fingerprint for butter produced from Alpine grazing systems [4], even though lipids are among the most variable components and are highly responsive to dietary and seasonal changes [5]. The FA profile of milk fat affects both nutritional and technological properties of butter, including melting and spreadability properties [5]. Dairy systems with high pasture inclusion may increase FA with a relevant healthy value for consumers, such as C18:1 *t*-11 (vaccenic acid) and C18:2 *c*-9, *t*-11 (isomer of conjugated linoleic acid, CLA), and branched-chain FA, while also influencing butter texture and spreadability [5]. The transition from cows’ winter feeding, typically based on conserved forages and concentrate supplementation, to fresh pasture grazing rapidly alters the FA composition of milk through changes in ruminal biohydrogenation pathways and mammary lipid metabolism [3,5]. Compared with winter diets, pasture-based feeding is consistently associated with increased concentrations of α-linolenic acid (C18:3 *n*-3), CLA, total polyunsaturated FA (PUFA), and odd- and branched-chain FA, thereby improving the fat nutritional quality in milk and dairy products [6,7]. Moreover, the dairy plant operative conditions might also contribute to additional variability through batch management, cream handling, churning and temperature settings, especially in the comparison between an industrial lowland dairy plant vs. a small-scale mountain dairy facility.

Taking into account this background, rapid, reliable and accurate analytical tools are needed to monitor butter quality. Although conventional wet-chemistry methods and gas chromatography (GC) for FA profiling provide accurate and comprehensive analyses, they are laborious, time-consuming, and require specialized laboratory facilities. In contrast, spectroscopy-based techniques offer a rapid, cost-effective, and environmentally friendly alternative. Near-infrared (NIR) spectroscopy has become a widely used alternative for food and dairy analysis because it is non-destructive, fast, chemical-free, and compatible with chemometric modeling [8,9]. NIR spectra contain information on overtone and combination bands of chemical bonds such as C–H, O–H, N–H, C=O, and C=C, which are highly relevant to fat-rich matrices such as butter [9]. When coupled with multivariate methods, NIR spectroscopy can support quantitative predictions and qualitative classification of dairy products, including composition, quality, authenticity, and seasonal variation [8,10].

Benchtop NIR instruments are generally preferred because they provide broad spectral ranges, high spectral resolution, stable acquisition conditions, and robust signal quality. However, handheld and portable NIR spectrometers are increasingly adopted because they enable rapid in situ, at-line, and on-farm analyses with minimal or no sample preparation, reducing analytical time and costs throughout the dairy supply chain [6]. Their portability makes them particularly attractive for decentralized quality control in small-scale dairy plants operating in rural areas, where access to specialized laboratories may be too expensive or impractical. Moreover, they support traceability and rapid decision-making at the butter plant or during product reception.

Despite their practical advantages, handheld NIR spectrometers differ from benchtop instruments in spectral range, wavelength sampling, spectral resolution, detector technology, optical configuration, and sample presentation geometry [6,8]. These differences may limit the direct transferability of calibration models, even between instruments of the same model and manufacturer [11], because wavelength shifts, photometric differences, detector response, spectral bandwidth, optical geometry, sample presentation, temperature, and environmental conditions can introduce systematic spectral discrepancies and reduce prediction robustness. Calibration transfer (CT) or model-maintenance strategies are therefore required to preserve prediction performance without developing a completely new calibration for each secondary instrument.

Several CT strategies have been proposed, differing in their assumptions and in the amount of information required from the target instrument. Classical standard-sample-based approaches, including direct standardization (DS) and piecewise direct standardization (PDS), require representative paired spectra from the primary and secondary instruments [12,13,14]. Although effective, this requirement may limit their practical implementation in decentralized applications involving portable or handheld instruments [13].

Therefore, more recent approaches include standard-light or standard-free strategies aimed at reducing dependence on repeated paired measurements. Among these, dynamic orthogonal projection (DOP) is particularly relevant because it removes spectral directions associated with instrumental or environmental variation while preserving chemically meaningful information for prediction [15]. Despite the extensive literature on CT, applications to fat-rich dairy products, especially butter, remain limited, particularly when the goal is to transfer calibrations from a high-performance benchtop NIR instrument to multiple handheld devices with different spectral characteristics. This represents a relevant gap because butter quality assessment requires methods that are both analytically reliable and operationally feasible across heterogeneous production systems. In the case of mountain butter samples, the analytical challenge is amplified by the variability due to season, cows’ feeding systems, butter plant location and processing. Therefore, CT should be tested not only under controlled conditions but also using representative samples and an appropriate hold-out validation design.

The present study investigated CT between a benchtop NIR instrument and three handheld NIR instruments for butter quality analysis. Based on this background, the aims of this study were: (i) to evaluate the feasibility of transferring NIR calibration models developed on a benchtop instrument to three handheld NIR instruments for butter quality assessment; (ii) to compare different CT strategies under realistic spectral differences among instruments; (iii) to assess the performance of transferred models using a hold-out validation set; and (iv) to determine whether handheld NIR spectroscopy, supported by appropriate CT methodology, can provide a rapid and practical tool for on-site butter chemical quality monitoring.

## 2. Materials and Methods

### 2.1. Samples, Spectral Acquisition and Reference Data

#### 2.1.1. Samples

Ninety-nine (*n* = 99) butter samples were collected from October 2023 to September 2024 following a monthly repeated-measures sampling protocol and classified into four groups according to the production system and milk origin:

- Lowland (*n* = 35): Monthly samples from industrial dairy plants processing milk from intensive dairy herds located in the Po Valley and fed maize silage-based total mixed rations (TMR);
- Mountain (*n* = 47): Monthly samples from industrial dairy plants located in mountain areas processing milk from dairy herds fed hay-based TMR;
- Alpine (*n* = 10): Summer samples from small-scale dairy plants processing milk from transhumant dairy herds grazing alpine pastures;
- Commercial (*n* = 7): Butter samples purchased from retail outlets with no information available on milk origin or production season.

After sample collection, they were stored at −20 °C until NIR and wet-chemistry analysis.

#### 2.1.2. NIR Spectral Acquisition

As stated in Figure 1, butter samples were analyzed using four NIR instruments: one benchtop NIR spectrometer, the FOSS DS2500 (FOSS Analytical A/S, Hillerød, Denmark; hereafter DS25), used as the master instrument, and three handheld NIR spectrometers used as target instruments. These latter were: Aurora (Grainit S.r.l., Padova, Italy), JDSU (JDS Uniphase Corporation, Milpitas, CA, USA), and ISC (Integrated Spectronics Pty Ltd., Castle Hill, Australia). For each sample, paired spectra were available from both instruments and were acquired in absorbance mode under comparable analytical conditions. The spectral matrices were aligned by sample identifier, so that each row of the master matrix corresponded to the same sample in the target matrix and in the reference chemical dataset.

Spectral ranges were 1100–2500, 0.5 nm for DS25; 950–1650, 2 nm for Aurora; 910–1670, 2 nm for JDSU; and 1102–1600, 2 nm for ISC, respectively. The common spectral range used for the CT procedure was 1102–1600 nm, with spectral variables arranged at 2 nm intervals (Figure 1). Before spectral acquisition, each sample was gently stirred and placed in a round quartz-window cup. The samples were maintained at 4 °C to minimize heating effects during scanning, which could arise from exposure to room temperature and lamp irradiation. To reduce potential bias associated with the acquisition sequence, the order of instrument use was randomly varied across samples.

#### 2.1.3. Reference Data

As reported in a previous study [16], moisture was determined by heating at 105 ± 2 °C to a constant weight, while fat content was determined using petroleum ether and diethyl ether (1:1, *v*/*v*), with the solvent then evaporated under an N_2_ flow at 50 °C. The fatty acid methyl esters (FAME) were prepared with an acid-catalyzed transesterification using a methanolic H_2_SO_4_ solution at 4% (*v*/*v*) overnight, and then after an extraction with n-heptane, the FA profile was determined via a 2010 Plus automated gas chromatograph (Shimadzu Italia, Milano, Italy) equipped with an Omegawax 250 capillary (30 m × 0.25 mm × 0.25 μm) column (Sigma-Aldrich, St. Louis, MO, USA) and a flame ionization detector. As carrier gas, helium was used at a constant flow rate of 0.8 mL min^−1^, and both the injector and detector were set at 255 °C. The oven program was set to an initial temperature of 80 °C, held for 2 min, ramped to 255 °C at 3.5 °C min^−1^, and held for 15 min. The FA were identified by comparing their retention times to those of authentic FAME using a 37-Component FAME mix standard (Supelco, Bellefonte, PA, USA). FA results were reported as g per kg of butter.

### 2.2. Data Splitting by Kennard–Stone Algorithm

Sample allocation was performed before any preprocessing, standardization, calibration or validation step. The same sample split was used for all preprocessing strategies, CT methods, and target instrument. The Kennard–Stone algorithm was applied to obtain representative subsets covering the spectral variability of the paired dataset. The matrix used for sample selection was derived from the master spectral matrices to account for transferability across target instruments.

The 99 samples were divided into four non-overlapping subsets: 20 paired samples for instrument standardization, 50 samples for CT, 10 samples for model updating, and 19 samples for hold-out validation. The hold-out validation samples were not used for preprocessing fitting, standardization, calibration, model updating, or Partial Least Squares (PLS)/DOP component selection. After these modeling steps were completed, they were used to evaluate and compare candidate configurations and to identify the best-performing validation models for descriptive comparison and reporting (Table 1).

### 2.3. Spectral Preprocessing Strategy and Parameter Grid

Spectral preprocessing was treated as an experimental factor. Available preprocessing options were: no preprocessing, standard normal variate (SNV) [17], multiplicative scatter correction (MSC) [18], detrending, Savitzky–Golay smoothing and Savitzky–Golay derivatives [19]. When smoothing or derivatives were selected, the corresponding parameter grids were defined a priori, including smoothing window length, polynomial order, derivative order and derivative gap. All selected combinations of preprocessing sequences and parameters were then tested systematically.

Preprocessing was applied after sample splitting and before CT. For each selected preprocessing configuration, the efficiency of preprocessing parameters was estimated in a cross-validation (CV) procedure using only non-validation samples, i.e., the standardization, calibration and update sets. These corresponded to 20 + 50 + 10 samples, accounting for available paired master–target information where applicable. The same fitted preprocessing pipeline was then applied to the hold-out validation spectra. Thus, the hold-out samples did not contribute to preprocessing fitting, while all candidate methods were subsequently evaluated under identical validation conditions.

### 2.4. Calibration Transfer Strategy

The CT methods tested in this study were selected to represent different levels of modeling complexity and different assumptions about the relationship between the master and target instruments. Spectral bias correction (SBC) was included as the simplest additive approach, assuming that the main instrumental difference can be approximated by a wavelength-specific offset. DS was included as a global multivariate transfer approach, in which a single transformation matrix is estimated to map spectra between instrumental domains. PDS was included as a local version of DS, designed to model wavelength-dependent relationships using neighboring spectral variables. Shenk–Westerhaus (S-W) standardization was applied as a wavelength-wise linear correction using paired master–target spectra. Finally, DOP was included as an orthogonal-projection approach to remove spectral directions associated with instrumental mismatch prior to PLS calibration.

In the present experimental design, CT was evaluated within a controlled paired-sample framework to enable fair comparisons among transfer methods. The same 20 standardization samples, measured on both the master and target instruments, were used to estimate the transfer relationship for methods requiring paired master–target information. In addition, 10 target-instrument spectra with reference values were included in the final calibration matrix as an update set. This update step corresponds to model inoculation or local calibration updating and was used to anchor the transferred calibration within the spectral domain of each handheld instrument. Therefore, the workflow should be interpreted as a standard-sample-based comparison of transfer algorithms supplemented by target-domain updating rather than as a fully standard-free transfer experiment.

CT was performed from the master to the target instrument to develop models applicable to samples measured directly on the target device. For standard transfer methods, including SBC, DS, PDS and S-W standardization, the 20 paired standardization samples were used to estimate the master-to-target correction. The resulting transfer function was then applied to the 50 master calibration spectra, producing target-compatible calibration spectra. These were combined with the 10 target update spectra measured directly on the handheld instrument, resulting in a final calibration matrix of 60 samples. The hold-out validation set consisted of 19 target-instrument spectra used to compare candidate configurations and calculate validation performance metrics.

#### 2.4.1. Spectral Bias Correction

SBC was used as the simplest standardization approach. For each wavelength, the mean difference between target and master spectra was calculated from the 20 paired samples of the standardization set. This wavelength-specific average difference was then added to the master spectra of the calibration set to shift them toward the target spectral response.

#### 2.4.2. Direct Standardization

DS [20] was used to estimate a global linear relationship between the master and target instruments. The transformation matrix was estimated from the 20 paired master–target spectra of the standardization set and then applied to the master spectra of the calibration set. Ridge regression was used for numerical stability when estimating the transformation matrix.

#### 2.4.3. Piecewise Direct Standardization

PDS [21] was applied as a local version of DS. For each target wavelength, a local regression model was fitted using a moving window of neighboring master wavelengths. The size of the PDS window was defined a priori by the operator, and all selected window sizes were tested as part of the method grid.

#### 2.4.4. Shenk–Westerhaus Standardization

The S-W standardization [22] was implemented as a wavelength-wise correction based on paired spectra acquired on the master and target instruments. For each wavelength, a linear correction between master and target absorbance values was estimated using the 20 standardization samples and then applied to the master calibration spectra.

#### 2.4.5. Dynamic Orthogonal Projection

DOP was applied to reduce spectral variation associated with instrumental differences between the master and target instruments. The method is based on the assumption that the difference between spectra acquired on two instruments contains a component related to the instrument, the optical configuration, or the measurement environment. This component can be estimated as an interference subspace and subsequently removed from the spectral data. Once this detrimental subspace is identified, spectra are projected onto its orthogonal complement, thereby reducing the influence of instrumental variation while retaining the spectral information relevant for predicting the reference variables [23].

Here, the projection matrix was estimated from the 20 paired master–target spectra of the standardization set after the selected transfer-preprocessing configuration. The difference matrix between the target and master spectra was computed and subjected to Principal Component Analysis (PCA) to identify orthogonal directions that describe master–target spectral mismatch. Candidate DOP models were generated by progressively removing an increasing number of these mismatch directions through orthogonal projection. For each candidate DOP model, the projection was applied to the 50 master calibration spectra, the 10 target update spectra, and the 19 hold-out target validation spectra. The final DOP–PLS calibration matrix therefore consisted of 50 projected master calibration spectra and 10 projected target update spectra, whereas the projected hold-out validation spectra did not contribute to estimation of the DOP projection or to DOP/PLS component selection. The number of DOP components was selected internally and was not optimized on the hold-out validation set. For each candidate number of DOP components, the projected calibration/update dataset was submitted to the PLS modeling stage. The selected PLS preprocessing configuration was fitted during the calibration procedure, and the number of PLS components was optimized by internal cross-validation. The DOP model yielding the lowest internal root mean square error in cross-validation (RMSECV) within each candidate preprocessing configuration was retained and subsequently evaluated on the hold-out validation set. Because this selection was performed separately for each response variable and PLS-preprocessing configuration, the selected number of DOP components was allowed to vary across models without using the hold-out validation set for DOP or PLS component selection.

Operationally, DOP differs from DS and PDS in that it does not attempt to transform each master spectrum into a direct numerical replica of its corresponding target spectrum. Instead, it removes spectral directions primarily associated with instrumental mismatch and calibrates in a projected space designed to be less sensitive to master–target differences. This feature makes DOP particularly suitable when instrumental differences are multivariate and distributed across the spectrum, as expected when transferring calibrations from benchtop NIR instruments to handheld devices with different spectral resolution, optical geometry, detector response, and signal-to-noise characteristics [23].

### 2.5. PLS Calibration and Component Selection

PLS regression was used to develop calibration models for each response variable, preprocessing configuration and CT method. The maximum number of latent variables to be tested was defined a priori by the operator. For each model, the optimal number of PLS components was selected via internal k-fold cross-validation on the calibration/update set, using RMSECV as the selection criterion.

For bias correction, DS, PDS and S-W standardization, the calibration matrix consisted of 50 transformed master spectra and 10 original target spectra, for a total of 60 samples. For DOP, the calibration matrix consisted of 50 projected master spectra and 10 projected target spectra. The same internal cross-validation strategy was used for all methods. The hold-out validation set was not used for PLS model fitting or PLS component selection.

### 2.6. Hold-Out Validation and Comparison of Models

All candidate models were evaluated using the 19 samples of the hold-out validation set. For the target transfer models, validation was performed on original target spectra processed with the same fitted preprocessing pipeline used for the calibration data. For DOP models, the target validation spectra were additionally projected using the internally selected DOP projection. For the master benchmark, an original calibration was developed using the 50 DS25 calibration spectra and the 10 DS25 spectra corresponding to the update samples, and validation was performed on the 19 original DS25 validation spectra.

All standardization methods were compared on a level playing field in terms of preprocessing. In other words, for a given preprocessing sequence and parameter setting, all transfer methods were evaluated using the same standardization, calibration, update, and validation samples. This design allowed the separate assessment of the effects of preprocessing and the CT method (Table 2). For each response variable and target instrument, we identified the candidate configuration with the lowest hold-out validation root mean square error of prediction (RMSEP) for descriptive comparison and reporting. Accordingly, the hold-out set was separate from model fitting and internal PLS/DOP component selection, but it was subsequently used for post hoc comparisons among fitted candidate configurations.

### 2.7. Performance Metrics and Result Tables

Model performance was summarized using the root mean square error of calibration (RMSEC), RMSECV, RMSEP, prediction bias, standard error of prediction after bias correction (SEP), coefficient of determination in prediction (R^2^_pred_), mean absolute error (MAE), ratio of performance to deviation (RPD), and slope and intercept of the regression between predicted and observed values.

### 2.8. Workflow Overview

The complete workflow, including Kennard–Stone splitting, preprocessing-grid definition, CT, internal component selection, hold-out validation, and comparative reporting, is summarized in Figure 2.

The algorithmic summary is as follows:

1. Align master spectra, target spectra and chemical reference data by sample identifier;
2. Perform Kennard–Stone splitting before preprocessing or model fitting;
3. Define the preprocessing sequences and parameter grids a priori;
4. For each preprocessing configuration, fit preprocessing parameters using only non-validation samples and apply the fitted pipeline to all required spectra;
5. For each transfer method, estimate the transfer function using the 20 paired standardization samples;
6. Build target-compatible calibration matrices using 50 transformed/projected master spectra and 10 original/projected target spectra;
7. Select PLS components by internal cross-validation for every model;
8. For DOP, select the number of DOP components by internal PLS cross-validation before hold-out validation;
9. Evaluate candidate models on the 19 hold-out validation samples after preprocessing fitting, transfer-function estimation, calibration/update, and internal PLS/DOP component selection have been completed;
10. Use hold-out validation metrics to identify the best-performing single configurations and, for the reduced ensemble, the top-ranked candidate configurations for descriptive comparison and reporting, and export the corresponding result tables.

### 2.9. Prediction-Based Evaluation of Calibration Transfer Consistency

In addition to conventional hold-out validation metrics calculated against reference values, we performed a prediction-based evaluation of CT consistency. This additional analysis was included because standard prediction errors, such as RMSEP, quantify the agreement between predicted and reference values and therefore include not only model-transfer error but also the uncertainty associated with the reference analytical method. Consequently, RMSEP remains essential for evaluating the accuracy of analytical predictions, but it does not isolate the instrumental component of CT. The prediction-based approach was used to assess whether the master and transferred target models produced comparable outputs for the same hold-out samples. For each response variable and validation sample, predictions were obtained from the DS25 benchmark model and from the transferred models developed for Aurora, JDSU, and ISC portable NIR devices. For each response variable and NIR device, the prediction set corresponding to the model with the lowest hold-out validation RMSEP was selected. These selected predictions were then used to calculate a robust consensus prediction, defined as the median of the available instrument-specific predictions for the same validation sample. The median was preferred to the mean because it is less sensitive to occasional outlying predictions from a single instrument. The selected predictions were subsequently used to calculate the prediction-consistency root mean square error (RMSE_consistency_) and the prediction bias of each target instrument relative to the DS25 benchmark [24].

For each response variable, prediction consistency across instruments was assessed on the external validation set by comparing each instrument’s predictions with a multi-instrument consensus prediction. For each validation sample, the consensus prediction was calculated as the median of the four predicted values obtained from the DS25 master instrument and the three target handheld instruments, namely Aurora, ISC and JDSU. The prediction-consistency RMSE_consistency_ for instrument (*j*) was then calculated as (Equation (1))

(1) $${RMSE}_{consistency,j}=\sqrt{\frac{1}{n}\sum_{i=1}^{n}{\left({\hat{y}}_{ij}-median\left({\hat{y}}_{i,master},{\hat{y}}_{i,target1},{\hat{y}}_{i,target2},{\hat{y}}_{i,target3}\right)\right)}^{2}}$$

where ${\hat{y}}_{ij}$ is the predicted value for sample *i* obtained with instrument *j*, and *n* is the number of external validation samples. In addition, the prediction bias of each target instrument relative to the DS25 benchmark was calculated as the mean difference between target and DS25 predictions on the same validation samples. These metrics were used only to evaluate the consistency of the transferred predictions across instruments, whereas RMSEP, R^2^_pred_, SEP, RPD, slope and intercept were retained as the primary indicators of agreement with the reference analytical values.

### 2.10. Software Implementation and Reproducibility

All data processing, spectral preprocessing, CT procedures, PLS modeling, cross-validation, external validation and results export were performed using a custom Python script developed for this study. The script was implemented in the Anaconda (version Navigator 2.6.6) within Python environment (version 3.9.21 64-bit) and run using Spyder 6.0.7.

The scripts used were SciPy [25] for spectral preprocessing procedures, including Savitzky–Golay smoothing, Savitzky–Golay derivatives [19], and detrending; and scikit-learn [26] for multivariate modeling and validation. Specifically, scikit-learn functions were used for Kennard–Stone-related scaling operations, PCA, PLS regression, ridge regression, linear regression, k-fold cross-validation, and calculation of validation statistics. The openpyxl package [27] was used to export the results to Excel workbooks (Microsoft Corporation, Microsoft Excel, Redmond, WA, USA, 2021) or CSV format.

To support data analysis, OpenAI ChatGPT (version 5.5) was utilized to assist in editing and refining the Python scripts. All AI-assisted code adjustments were critically reviewed, verified, and validated by the authors to ensure technical accuracy. For full transparency and reproducibility, the developed Python script is available as Supplementary Materials (Supplementary Files S1–S4). Supplementary File S1 contains the Python code for the complete CT and PLS modeling workflow; Supplementary File S2 provides the code for the reduced top-five ensemble analysis; Supplementary File S3 contains the Python code used to generate the official Kennard–Stone sample split from the DS25 master spectra; and Supplementary File S4 reports the corresponding fixed allocation of the 99 samples to the standardization, calibration, update, and hold-out validation subsets.

### 2.11. Reduced Top-Five Ensemble Workflow

After exhaustive CT screening, a reduced-ensemble workflow was evaluated to determine whether a small number of robust modeling configurations could support future model updating and deployment on additional handheld instruments. This analysis was performed separately for each target instrument. Only transferred models were considered for the ensemble selection, whereas the original master benchmark was excluded. For each response variable and target instrument, all candidate CT configurations generated during screening were ranked according to hold-out validation performance.

Each configuration represented a complete modeling workflow, including transfer preprocessing, CT method, PLS preprocessing, and the number of PLS components selected by internal cross-validation. When applicable, the configuration also included the PDS window size or the number of DOP components. The five best configurations were selected using a composite ranking approach, conceptually related to the sum of ranking differences (SRD) procedure used in chemometrics to compare models or methods fairly across multiple performance criteria [28]. For each response variable and target instrument, transferred models were ranked according to five criteria: RMSEP, R^2^_pred_, absolute bias, RPD, and slope. For each criterion, the best-performing model received rank 1, thus favoring solutions with lower RMSEP and absolute bias, higher R^2^_pred_ and RPD, and a slope closer to 1.

The ranks were summed for each model, and the five configurations with the lowest total scores were retained. This approach promotes configurations that show balanced performance across accuracy, goodness of fit, bias, relative predictive ability, and linearity of prediction rather than excelling in a single criterion. For the ensemble prediction, the five selected configurations were applied to the hold-out validation samples. For each validation sample, the final prediction was calculated as the arithmetic mean of the five individual predictions. The standard deviation was also calculated as an indicator of agreement among the selected configurations and, consequently, of prediction stability. The performance of the reduced ensemble workflow was evaluated by comparing the averaged predicted values with the corresponding reference values on the hold-out validation set. The following metrics were recalculated from the averaged predictions: ensemble RMSEP (RMSEP_ens)_, ensemble R^2^ of prediction (R^2^_pred,ens_), and ensemble RPD (RPD_ens_). Importantly, ensemble performance was calculated from the averaged predictions and not by averaging the validation statistics of the five individual models. This strategy, which averages predictions from the five top-ranked configurations rather than selecting a single best model, follows established chemometric practice. Averaging or stacking multiple PLS regression models has been shown to improve robustness and prediction stability, particularly when model dimensionality or configuration choice is uncertain [29].

For each response variable and target instrument, the ensemble result was compared with the best single configuration in the selected top-five set using RMSEP and R^2^_pred_. The percentage change in RMSEP was calculated to evaluate whether the ensemble improved, maintained, or worsened prediction performance.

Because the same hold-out set was used both to rank the candidate configurations and to evaluate the averaged predictions of the resulting ensemble, the ensemble metrics—RMSEP_ens_, R^2^_pred,ens_, and RPD_ens_—should be interpreted as exploratory and descriptive comparisons within the present dataset. They do not represent fully independent estimates of generalization performance and should not be extrapolated to completely unseen future production batches.

## 3. Results

The preprocessing, cross-validation, and PLS parameters adopted are summarized in Table 3.

### 3.1. Main Results for Calibration Transfer-PLS Procedure

The Pearson correlation coefficient (r) between instruments was calculated, and the average value for paired instruments is reported in Table 4. The pairwise Pearson coefficient of correlation (r) map drawn by wavelengths is reported in Figure 3.

The CT procedure was evaluated for three handheld NIR instruments, namely Aurora, JDSU, and ISC, using the DS25 benchtop system as the master instrument, within the common spectral band and resolution (1102–1600, 2 nm). The best-performing transferred configuration for descriptive reporting was identified from the hold-out-validation results as the configuration with the lowest RMSEP. When a single model had to be retained for tabulation, the model with the lowest RMSEP was selected and then interpreted together with the remaining validation statistics, including prediction bias, SEP, R^2^_pred_, MAE, RPD, slope, and intercept. On this basis, model performance differed among target instruments. Across the 32 response variables, Aurora and JDSU showed the most favorable overall behavior, with mean RMSEP values of 0.29 and 0.29, mean R^2^_pred_ values of 0.62 and 0.57, and mean RPD values of 1.96 and 1.89, respectively. ISC showed lower and more variable performance, with a mean RMSEP of 0.41, a mean R^2^_pred_ of 0.35, and a mean RPD of 1.36 (Table 5).

Overall, Table 6 shows that the optimal transfer strategy was strongly instrument- and response-dependent. DOP and SBC provided the most favorable balance between selection frequency and prediction performance for Aurora and JDSU, whereas ISC showed a less stable pattern, with PDS most frequently selected but generally lower mean R^2^_pred_ and RPD, suggesting weaker and more variable transfer robustness for this instrument.

When the best transferred handheld instrument was selected for each response variable, Aurora provided the lowest-RMSEP transferred model for 18 of 32 variables, JDSU for 12, and ISC for two. For 30 of 32 response variables, the best transferred model produced a lower RMSEP than the corresponding DS25 master benchmark. In saturated fatty acids (SFA), the RMSEP was slightly higher than the master benchmark but remained within +10% (Table S1). Only C16:0 showed an RMSEP increase greater than 10% relative to the master benchmark. These results indicate that the combination of transfer standardization, target-instrument updating and independent PLS preprocessing was generally effective in preserving the predictive information required for hold-out validation.

The most accurate transferred models were generally obtained for the main compositional traits and the most abundant fatty-acid groups. High predictive ability was observed for moisture, fat content, SFA and monounsaturated fatty acids (MUFA), several medium- and long-chain FA, PUFA-related traits, and CLA. Conversely, lower and more variable performances were observed for several minor FA and low-variance traits, such as C15:0, C16:1, C17:0, C17:1, C20:0, C20:4 *n*-6, and the *n*-6/*n*-3 ratio. For these responses, the limited concentration range in the hold-out validation set likely constrained the achievable R^2^_pred_ and RPD, even though the absolute RMSEP remained low.

Aurora- and JDSU-handled NIR devices provided the most robust overall performance, whereas ISC achieved acceptable results only for selected traits and showed weaker average robustness. The optimal transfer algorithm depended on both the target instrument and the response variable, confirming that no single CT strategy was universally optimal. For Aurora, the selected models were distributed across DOP, SBC, PDS, S-W standardization, and DS. For JDSU, DOP and PDS were the most frequently selected approaches, followed by S-W, DS, and SBC. For ISC, PDS was selected most frequently, followed by DOP and S-W standardization, although the overall performance was more heterogeneous than for Aurora and JDSU. Therefore, there is a need to optimize CT separately for each instrument–response combination rather than applying a fixed transfer algorithm across all responses.

### 3.2. Main Results for Prediction-Based Consistency

Prediction-based consistency metrics were calculated to complement the hold-out validation statistics and to evaluate whether the transferred models produced outputs comparable with those of the DS25 benchmark and the other handheld instruments (Table S2). This analysis focused on the agreement among instrument-specific predictions for the same validation samples and was therefore not affected by the uncertainty of the reference analytical method.

The prediction-consistency analysis showed that the transferred models not only differed in their agreement with reference values but also in their ability to reproduce the prediction behavior observed across instruments. For most response variables, the target instrument with the lowest RMSEP also had a low RMSE_consistency_, indicating that the best transferred models were generally accurate relative to the reference method and consistent with the multi-instrument prediction consensus.

For some response variables, however, discrepancies were observed between RMSEP and prediction-consistency RMSE. In these cases, a transferred model could be relatively close to the reference values but less aligned with the DS25/multi-instrument prediction consensus, or, conversely, could be highly consistent across instruments despite only a moderate RMSEP. This distinction suggests that prediction accuracy and transfer consistency provide complementary information.

### 3.3. Reduced Top-Five Ensemble Workflow Across Target Instruments

The reduced top-five ensemble workflow was evaluated for the three DS25-to-handheld CT pathways: DS25-to-Aurora, DS25-to-ISC and DS25-to-JDSU. For each target instrument, the five most balanced transferred configurations were selected for each response variable using the composite ranking criterion, and the final prediction was obtained by averaging the five individual predictions. The ensemble workflow exhibited varying performance levels across target instruments. The best overall results were obtained for Aurora and JDSU, whereas ISC showed more limited predictive ability (Table 7). For Aurora, 13 out of 32 response variables achieved R^2^_pred,ens_ ≥ 0.70, and 19 achieved R^2^_pred,ens_ ≥ 0.50. For JDSU, 13 variables achieved R^2^_pred,ens_ ≥ 0.70, and 18 achieved R^2^_pred,ens_ ≥ 0.50. In contrast, for ISC, only two variables achieved R^2^_pred,ens_ ≥ 0.70, and six achieved R^2^_pred,ens_ ≥ 0.50. The ensemble also showed a generally favorable comparison, with the best single configuration retained within the top-five set. For Aurora, the ensemble reduced RMSEP_ens_ across 22 of 32 variables, with an average change of −3.32% and a median change of −1.45%. No variable showed an increase in RMSEP greater than 5%. For ISC, the ensemble reduced RMSEP_ens_ for 19 of 32 variables, with an average change of −2.79% and a median change of −0.58%, although two variables showed an increase in RMSEP_ens_ greater than 5%. For JDSU, the ensemble reduced RMSEP_ens_ in 17 out of 32 variables, with an average RMSEP_ens_ change of −1.51% and a median change of −0.15%; only one variable showed an RMSEP_ens_ increase greater than 5%.

Overall, Aurora and JDSU showed the most promising transfer results. Aurora had the highest mean R^2^_pred,ens_ across response variables, whereas JDSU showed a similar number of variables with good predictive ability and a high median R^2^_pred,ens_. ISC showed lower average and median R^2^_pred,ens_ values, indicating that the DS25-to-ISC transfer was more challenging. The best-performing variables differed slightly among target instruments. For Aurora, the strongest ensemble performances were observed for C18:1 *n*-11 trans-vaccenic acid, moisture, fat, SFA, C14:0, CLA and C10:0. For JDSU, the highest R^2^_pred,ens_ values were obtained for MUFA, moisture, fat, C18:1 *n*-9 cis/trans, C18:1 *n*-11 trans-vaccenic acid and SFA. For ISC, the best results were obtained for moisture, fat, SFA, C14:0, C16:0, and CLA, although the corresponding R^2^_pred,ens_ values were generally lower than those observed for Aurora and JDSU.

## 4. Discussion

The FA profile of milk fat strongly influences both the nutritional value and the technological properties of butter, including its melting behavior and spreadability [4]. While the FA composition of butter is largely determined by biological and production-related factors, particularly cow feeding and management practices, its total fat content depends primarily on butter-making processes, especially the efficiency of moisture removal during churning and working [30]. The growing demand for rapid, reliable, and portable analytical methods has increased interest in handheld NIR spectroscopy as a tool for on-site butter quality assessment outside specialized laboratories.

In this context, the present study evaluated the transferability of calibration models developed on a DS25 benchtop spectrometer to three handheld NIR instruments (Aurora, JDSU, and ISC). The raw spectra acquired with the handheld devices showed a high degree of similarity to those collected with the DS25, with Pearson correlation coefficients of 0.92, 0.94, and 0.98 for ISC, Aurora, and JDSU, respectively. The highest spectral agreement was observed in the 1100–1200 nm and 1350–1400 nm wavelength regions, which are dominated by lipid-related C–H overtone and combination absorptions in edible fats and oils [31]. Notably, the 1100–1200 nm region is mainly associated with second overtones of C–H stretching vibrations from –CH_2_, –CH_3_ and –CH=CH– groups, whereas the 1350–1400 nm region falls within the 1350–1430 nm interval attributed to C–H combination bands of –CH_2_ and –CH_3_ groups [32]. Therefore, the higher inter-instrument correlations in these regions likely reflect shared spectral information from lipid chains, particularly methylene and methyl groups, which are abundant in butter fat. This interpretation is consistent with the triglyceride-rich nature of butter and its high proportion of SFA, particularly palmitic (C16:0), myristic (C14:0) and stearic (C18:0) acids. However, spectral bands excluded by the present trial, around 1720, 1760, and 2148 nm, have been reported to discriminate between samples rich in SFA and unsaturated fatty acids (PUFA and MUFA), and these should be interpreted as discriminant lipid bands rather than exclusive markers of SFA [32].

Different combinations of spectral preprocessing, CT algorithms, and limited target-domain updating were tested to obtain target-compatible calibration models that predict validation samples measured directly on each NIR handheld device. This design reflects a practical deployment scenario in which existing benchtop calibrations are reused on portable instruments, but where direct model transfer cannot be assumed because benchtop and handheld devices may differ in spectral range, resolution, optical configuration and detector response. The need for CT was consistent with previous studies showing that models developed on one NIR instrument, including handheld devices, may yield biased predictions when applied directly to spectra acquired on secondary instruments [33]. In the present study, this issue was particularly relevant because the transfer was performed from a benchtop system to portable devices with different spectral characteristics. Therefore, the objective was not simply to compare instruments but to evaluate whether appropriate standardization and updating strategies could preserve the predictive information required for butter quality assessment in the handheld spectral domain.

Spectral preprocessing played an important role in the transfer workflow because it can reduce unwanted variation related to scattering, baseline shifts and noise [34]. However, preprocessing cannot be considered independently from CT. A preprocessing strategy that improves standalone calibration may not necessarily improve transfer performance, especially when instruments differ markedly in spectral resolution, wavelength registration or optical configuration [35,36]. For this reason, preprocessing was optimized separately within the transfer and PLS modeling steps, allowing each CT strategy to be evaluated under its most suitable conditions.

The comparison among CT approaches confirmed that no single method was universally optimal across instruments and response variables. This result is expected because each transfer method relies on different assumptions about the structure of inter-instrument differences. Simple bias or slope/bias correction is attractive because it is easy to implement and interpret, but it is mainly suited to situations where instruments are already well aligned, and the residual discrepancy is dominated by offset or slope effects [11,13]. DS models the relationship between instruments using the complete multivariate spectral signal, whereas PDS performs local wavelength-dependent standardization and remains one of the most widely used approaches in NIR CT [12,13,14,15]. However, PDS requires selecting local modeling parameters and may be less robust when instruments differ substantially in spectral range, resolution, or signal quality [13,15]. S-W standardization represents a simpler historical approach to NIR standardization [12], whereas DOP follows a different logic, identifying and removing spectral subspaces associated with instrumental mismatch rather than directly mapping one spectrum onto another [15]. The inclusion of DOP was therefore relevant in the present benchtop-to-handheld setting, where spectral differences were not expected to be limited to simple wavelength-wise offsets. At the same time, classical standard-sample-based methods such as DS and PDS provided a rigorous benchmark because paired spectra were available for the same samples measured on the master and target instruments. The study was not designed as a fully standard-free transfer, but the results provide a useful basis for future work aimed at reducing dependence on paired standardization samples. Possible extensions include domain-invariant PLS approaches, stable-wavelength selection strategies, or DOP-based workflows relying on occasional target-domain control samples rather than complete paired standardization sets. The limited target-domain update was an important component of the workflow. Adding a small number of target-instrument spectra with reference values to the transferred calibration set helped adapt the models to the handheld domain and reflects a realistic compromise between full recalibration and direct model transfer. This step is critically relevant for on-site butter quality control, where handheld instruments are attractive because they can be used outside specialized laboratories, but where local validation remains necessary to detect matrix, seasonal, or instrument-specific shifts.

### 4.1. Transfer-PLS Procedure

Model performance was evaluated using commonly used thresholds for NIR applications. RPD values greater than 3 are generally considered indicative of good predictive ability for quantitative analyses. As a reference framework, the classification proposed by [37,38] for forages and feeds was adopted: RPD values were considered excellent (>4.1; suitable for any application), very good (3.5–4.0; suitable for process control), good (3.0–3.4; suitable for quality control), fair (2.5–2.9; suitable only for screening), poor (2.0–2.4; suitable only for rough screening), or very poor (0.0–1.9; not recommended). Similarly, R^2^ values were interpreted to classify model performance for compound feedstuffs as excellent (R^2^ ≥ 0.90), good (0.70 ≤ R^2^ < 0.90), or having limited predictive ability (0.50 ≤ R^2^ < 0.70) [39]. However, the combined use of RPD and R^2^ has been considered partly redundant because RPD is mathematically related to R^2^ and therefore provides largely overlapping information on model fit [40]. Moreover, RPD thresholds are matrix-dependent and inherently relative: fixed classification schemes lack a rigorous statistical basis, and their application should be contextualized accordingly [40]. This consideration is highly relevant for butter and dairy products, whose composition is intrinsically variable due to the combined influence of season, feeding regime, geographical origin, and production system [41,42]. Natural conditions of the production area directly affect the quality of dairy products, with quality varying across sub-areas as differences in forage composition and cow feeding rations are transmitted through milk to the final product [43]. In this context, the ability of NIR-based models to discriminate and quantify compositional attributes depends directly on how well the calibration dataset captures this natural variability. Models based on NIR spectra for predicting trans FA in butter have achieved validation coefficients of determination of approximately 0.91–0.95, demonstrating that NIR spectroscopy can reliably capture the compositional fingerprint of butter fat. However, the accuracy of such models is inherently constrained by sample heterogeneity and the breadth of compositional variation represented in the calibration set, underscoring the importance of robust model evaluation beyond single scalar performance metrics such as RPD [44].

Broadly, previous studies have confirmed the potential of NIR spectroscopy for estimating lipid-related traits in dairy matrices, although most applications have focused either on a limited number of variables or on a single instrument. In butter, Heussen et al. (2007) [45] reported good NIR performance for butter fat content and C4:0, with RMSEP values of 4.3–8.2% and 0.33–0.38% *w*/*w*, respectively, supporting the ability of NIR spectra to capture information related to milk fat authenticity markers. In cheese, González-Martín et al. (2020) [46] showed that NIR spectroscopy can predict several FA, especially when intact cheese was analyzed directly with a fiber-optic probe, with RPD values generally ranging from screening to good predictive ability for major and nutritionally relevant FA. The relevance of this comparison is further supported by studies showing that FA profiles are largely preserved from milk to cheese during processing and ripening while still reflecting diet and seasonal effects. The improved performance observed for major compositional traits and abundant FA groups is also biologically and analytically plausible. Milk and dairy fat composition is strongly affected by diet, season, pasture access, and management, and major FA groups such as SFA, MUFA, PUFA, C16:0, and C18:1 generally exhibit greater natural variation than low-abundance individual FA [47]. Wider variation improves the attainable R^2^_pred_ and RPD, whereas narrow concentration ranges and low analytical variability may limit external validation performance, even when RMSEP is numerically small. Therefore, the weaker performance for some minor FA should not be interpreted only as a failure of CT but also as a consequence of the intrinsic difficulty of predicting low-variance traits by NIR spectroscopy. Against this background, the present study extends prior evidence by evaluating not only the prediction of lipid-related traits but also the transferability of these predictions from a benchtop instrument to multiple handheld NIR devices. Although performance varied across individual FA, the transferred handheld models achieved good predictive ability for several major traits and FA groups, including fat, SFA, MUFA, CLA, and selected C18-chain-based FA. Therefore, our findings are meaningful because they indicate that handheld NIR spectroscopy can retain useful predictive information for butter FA profiling after CT, a more demanding scenario than single-instrument calibration in butter or cheese.

Workman (2018) defined CT as the attempt to apply a single spectral database and calibration model across two or more instruments while retaining statistical accuracy and precision, but also emphasized that identical predictions across instruments remain difficult to achieve in practice because of instrument, sampling, and reference-method uncertainty [12]. The present results confirm that the transfer of DS25-based PLS calibrations to handheld NIR instruments is feasible for butter compositional and FA profiling but also show that transfer performance was strongly dependent on the target instrument, response variable, and transfer strategy. This pattern is consistent with the general principle that CT is not a simple model-copying procedure: even instruments designed for similar analytical purposes may differ in spectral range, resolution, detector response, optical configuration, wavelength registration, sample presentation geometry, and environmental sensitivity, all of which may affect the prediction performance of a model developed on a master instrument.

The better predictive performance observed for Aurora and JDSU compared with ISC should not be interpreted as evidence of intrinsic hardware superiority. Within the common 1102–1600 nm spectral range, the raw spectral similarity with the DS25 was highest for JDSU (r = 0.98), followed by Aurora (r = 0.94) and ISC (r = 0.92), suggesting a greater master–target spectral mismatch for ISC. Because all target instruments were evaluated using the same sample allocation, preprocessing framework, target-domain updating strategy, and multiple CT methods, the weaker ISC performance is unlikely to reflect the failure of a single transfer algorithm. Restricting all instruments to the same nominal wavelength range and 2 nm spectral grid harmonized spectral coverage but did not necessarily equalize the quality or origin of the spectral information. Rather, it may result from instrument-specific spectral characteristics and their interaction with the transfer procedure. Potential contributing factors include detector response, optical configuration, wavelength registration, signal-to-noise properties, and sample-interface effects; however, these characteristics were not independently quantified in the present study. Therefore, the relative contributions of hardware and CT interactions cannot be disentangled from the current experimental design. However, optical geometry, detector response, and signal-to-noise ratio were not independently characterized in the present study. Therefore, the relative contribution of these potential hardware-related factors cannot be determined, and they should be regarded as plausible explanations rather than demonstrated causes of the weaker ISC performance. This confirms that handheld NIR systems can provide useful quantitative information, but their performance cannot be assumed to be equivalent across NIR devices. This point is notably relevant because portable and handheld NIR instruments are increasingly promoted for rapid, non-destructive, in situ and online applications, but their miniaturized optical designs and reduced wavelength ranges may introduce device-specific limitations that require chemometric correction and validation [9]. This result agrees with the CT literature, which indicates that method suitability depends on the structure and linearity of the residual differences between master and target instruments [11]. The fact that different transfer algorithms were selected for different responses suggests that the spectral distortions affecting the handheld instruments were not uniform across the whole spectral space or across all chemical traits. This is expected for butter FA prediction, where some response variables are associated with stronger and broader spectral information, while minor FA may depend on weaker, indirect or highly collinear spectral signals.

### 4.2. Prediction-Based Consistency

The prediction-based consistency analysis provided complementary information to the conventional validation statistics. While RMSEP, SEP, R^2^_pred_, RPD, slope and intercept remain essential to evaluate agreement with reference analytical values, they do not isolate the instrumental component of CT. Indeed, RMSEP includes not only the error introduced by model transfer and spectral differences between instruments but also the uncertainty of the reference analytical method. Eskildsen et al. (2016) [24] explicitly highlighted that evaluating transfer success only by comparing predictions with reference values may be misleading because reference uncertainty can make a transferred model appear poor even when different instruments produce very similar predictions for the same samples. This is especially relevant in multi-instrument applications, where the objective of CT is not only to preserve agreement with the reference method but also to ensure consistency of predictions across instruments.

For this reason, comparing instrument-specific predictions with a consensus prediction is particularly useful in the present study. The aim of CT is not only to minimize the error against laboratory reference values but also to obtain comparable predictions when the same sample is analyzed using the master and target instruments. This approach is consistent with the theoretical definition of successful CT, in which a model developed on one instrument should provide statistically comparable predictions when applied to other instruments [12].

In the present dataset, Aurora and JDSU showed the highest prediction consistency relative to the multi-instrument consensus, including DS25. The mean prediction-consistency RMSE was slightly lower for Aurora than for JDSU (approximately 0.162 vs. 0.159), whereas ISC showed a higher mean value of approximately 0.283. However, when consistency was evaluated separately for each response variable, JDSU was the most frequently selected target instrument, showing the lowest RMSE_consistency_ for 20 of 32 variables, followed by Aurora for 10 variables and ISC for two variables.

The bias analysis relative to the DS25 benchmark provided an additional interpretation of the transfer pattern. Aurora and JDSU showed relatively low mean absolute biases relative to DS25, at approximately 0.074 and 0.080, respectively, whereas ISC showed a higher mean absolute bias of approximately 0.112. This suggests that ISC predictions were, on average, less accurate in external hold-out validation statistics and less aligned with the DS25 prediction scale. This is a key result because a transferred model may show an acceptable RMSEP for some traits but still present a systematic prediction offset relative to the master instrument, which can be problematic in routine multi-instrument monitoring.

The largest inconsistencies were observed for broader fat-related chemical groups such as SFA, MUFA, *n*-6/*n*-3 ratio and/or specific FA like C16:0. Zhao et al. (2015) [48] highlighted that milk fat contains more than 400 FA and that naturally occurring trans FA showed wide concentration ranges, indicating substantial compositional variability in dairy lipid fractions. These lipidic fractions are characterized by relatively high biological variability [47] and strong spectral contribution but also by high collinearity and dependence on lipid matrix structure. In practical terms, this means that the same traits that are often easier to predict by NIR because they vary widely may also amplify residual differences between instruments when spectral transfer is imperfect. Conversely, FA at low concentrations showed small absolute prediction errors, but this should be interpreted cautiously because low-concentration ranges naturally constrain the magnitude of prediction differences. This interpretation is consistent with previous findings [49], which reported low SEP values for several minor FA, despite poor or only approximate predictive performance, and concluded that prediction quality decreased when FA were present in low to very low concentrations. Taken together, the prediction-based analysis strengthens the interpretation of the CT results. RMSEP-based validation identified the best analytical prediction performance relative to reference values, whereas prediction-consistency RMSE assessed whether the transferred instruments behaved similarly to the DS25 benchmark and to one another. Therefore, the two approaches address different but complementary questions: conventional validation assesses analytical accuracy, while prediction-based consistency assesses inter-instrument agreement. The combination of both criteria is especially relevant for the deployment of handheld NIR instruments in decentralized quality-control networks, where the objective is not only to obtain accurate predictions but also to ensure that different instruments provide comparable decisions for the same butter samples.

Overall, prediction-based consistency provided complementary information to reference-based validation by helping distinguish limitations related to instrumental transfer from those associated with the intrinsic predictability of a given trait or with reference-method uncertainty. For example, moderate RMSEP combined with good agreement among instrument-specific predictions may indicate that prediction performance is constrained mainly by the trait itself or by the reference method, whereas poor agreement between DS25 and handheld predictions is more suggestive of incomplete compensation of instrumental differences. Thus, the combined interpretation of reference-based and prediction-based metrics provides a more comprehensive assessment of CT performance. This is particularly relevant for decentralized multi-instrument applications, where transferred models should provide not only accurate predictions relative to reference values but also comparable outputs across instruments [24].

### 4.3. Instrument-Dependent Performance of the Reduced Ensemble Strategy

While Lesnoff et al. (2022) [29] focused specifically on averaging PLS models differing only in their number of latent variables, the present ensemble extends this principle to a broader space of modeling choices, including transfer preprocessing, CT method, and PLS preprocessing, which are selected jointly through a composite multi-criterion ranking procedure.

The reduced top-five ensemble workflow was developed to provide a more practical alternative to the exhaustive evaluation of all combinations of preprocessing, CT, and PLS. This approach is consistent with previous chemometric evidence showing that averaging the predictions of multiple PLS regression models, rather than relying on a single optimally tuned model, tends to improve robustness and reduce dependence on a specific model configuration, even when a simple uniform-weight average is used [29]. The results indicate that this approach can reduce dependence on a single selected model while maintaining, and in several cases improving, hold-out prediction performance within the present dataset.

The ensemble strategy was most effective for Aurora and JDSU, whereas its benefit was more limited for ISC. This suggests that averaging a small set of well-performing transferred configurations can improve prediction robustness when the underlying models already retain adequate predictive information. Conversely, ensemble averaging cannot fully compensate for limited master–target compatibility, as observed for ISC. The more favorable results for moisture, fat, major FA groups, and selected individual FA may reflect stronger spectral information and/or greater reference variability for these traits, which can support more stable CT. Overall, the effectiveness of the ensemble depends not only on the averaging procedure itself but also on the quality and stability of the transferred models entering the ensemble.

The comparison with the best selected individual configurations indicates that the ensemble primarily improved prediction stability rather than systematically increasing absolute accuracy. For Aurora, averaging the selected configurations generally improved performance without relevant deterioration, whereas for JDSU, the ensemble behaved mainly as a conservative strategy, maintaining performance close to that of the best selected configuration. For ISC, averaging frequently reduced prediction error but could not overcome the lower predictive performance of the underlying transferred models. Overall, these findings suggest that the benefit of ensemble averaging depends strongly on the quality of the candidate models entering the ensemble. The comparison with the best selected individual configurations is particularly relevant. For Aurora, the ensemble improved RMSEP_ens_ across most variables and did not cause any relevant RMSEP_ens_ to worsen by more than 5%, suggesting that averaging the five selected predictions provided a robust operational solution. For JDSU, the ensemble was slightly less often better than the best selected single model, but it remained highly stable: 27 out of 32 variables were within ±5% RMSEP relative to the best selected single configuration, and only one variable showed a worsening greater than 5%. This suggests that, for JDSU, the ensemble may be especially useful as a conservative, stable prediction strategy rather than as a systematic means to improve absolute accuracy. For ISC, the ensemble also frequently reduced RMSEP, but the lower absolute R^2^_pred_ and RPD values indicate that the main limitation was not the ensemble procedure itself but the quality of the calibration and transfers achievable for this target instrument.

However, the ensemble workflow should not be interpreted as a replacement for proper CT validation. Instead, it represents an operational layer that can be applied after model screening to simplify future use. Once balanced configurations have been identified, only a restricted set of workflows needs to be retained for routine prediction or model updating, reducing dependence on a single configuration that may be sensitive to the validation split or preprocessing choice. The instrument-dependent behavior observed here is consistent with previous evidence from PLS model averaging [29] and with broader findings showing that differences among NIR instrument classes can influence model accuracy, sensitivity, and robustness [50].

Because preprocessing optimization, CT, and target-domain updating were evaluated as components of an integrated workflow, the present design does not allow their individual contributions to prediction improvement to be disentangled. A factorial ablation analysis in which these components are systematically introduced or removed while keeping all remaining modeling choices fixed would be valuable in future studies.

A limitation of the present study is the relatively small dataset and, in particular, the reliance on a single 19-sample hold-out validation split, despite the large number of preprocessing, CT methods, and modeling configurations explored. Although Kennard–Stone selection was used to obtain spectrally representative subsets, and the same sample allocation was maintained across all methods and instruments to ensure a fair within-study comparison, the ranking of candidate configurations may still be sensitive to this specific sample allocation. In addition, the hold-out validation set was used both to compare candidate configurations and to report the performance of the best-performing configurations and the reduced top-five ensemble. Accordingly, these validation results should be interpreted as exploratory estimates of transfer performance rather than as fully independent measures of generalizability since using the same hold-out set for model comparison and performance assessment may lead to over-optimistic estimates of performance on fully independent external data. Confirmation on a larger dataset—preferably including an additional independent validation cohort and/or repeated evaluation across alternative data splits—will be required to establish the robustness of the identified instrument–response–transfer combinations.

A further limitation is that the temporal stability of the transferred models and transfer coefficients was not evaluated through repeated spectral measurements. Such an assessment would be particularly relevant for routine industrial deployment, where instrumental response may vary over time. Future studies should therefore include repeated measurements of the same samples over defined time intervals to evaluate the short- and long-term stability of CT methods performance.

## 5. Conclusions

This study showed that calibration models developed on a benchtop NIR instrument can be transferred to handheld devices for butter composition and FA profiling when appropriate preprocessing, CT, and limited target-domain updating strategies are combined. Transfer performance was dependent on the target instrument and response variable, and no single CT method was universally optimal. The reduced top-five ensemble provided a practical approach for simplifying model deployment while maintaining stable predictive performance.

Overall, the findings support the potential of handheld NIR spectroscopy for decentralized butter quality monitoring while emphasizing the need for target-instrument validation and representative calibration and validation datasets.

Given the limited sample size and the use of a single hold-out split for both comparative model ranking and ensemble evaluation, the present findings—and particularly the reported ensemble metrics—should be considered exploratory and descriptive rather than definitive estimates of generalizability to unseen future batches. Future studies should confirm the results using larger and temporally independent datasets, assess the long-term stability of transferred calibrations under routine operating conditions, and investigate standard-light or standard-free CT strategies to reduce dependence on repeated paired measurements.

## Figures and Tables

**Figure 1:**
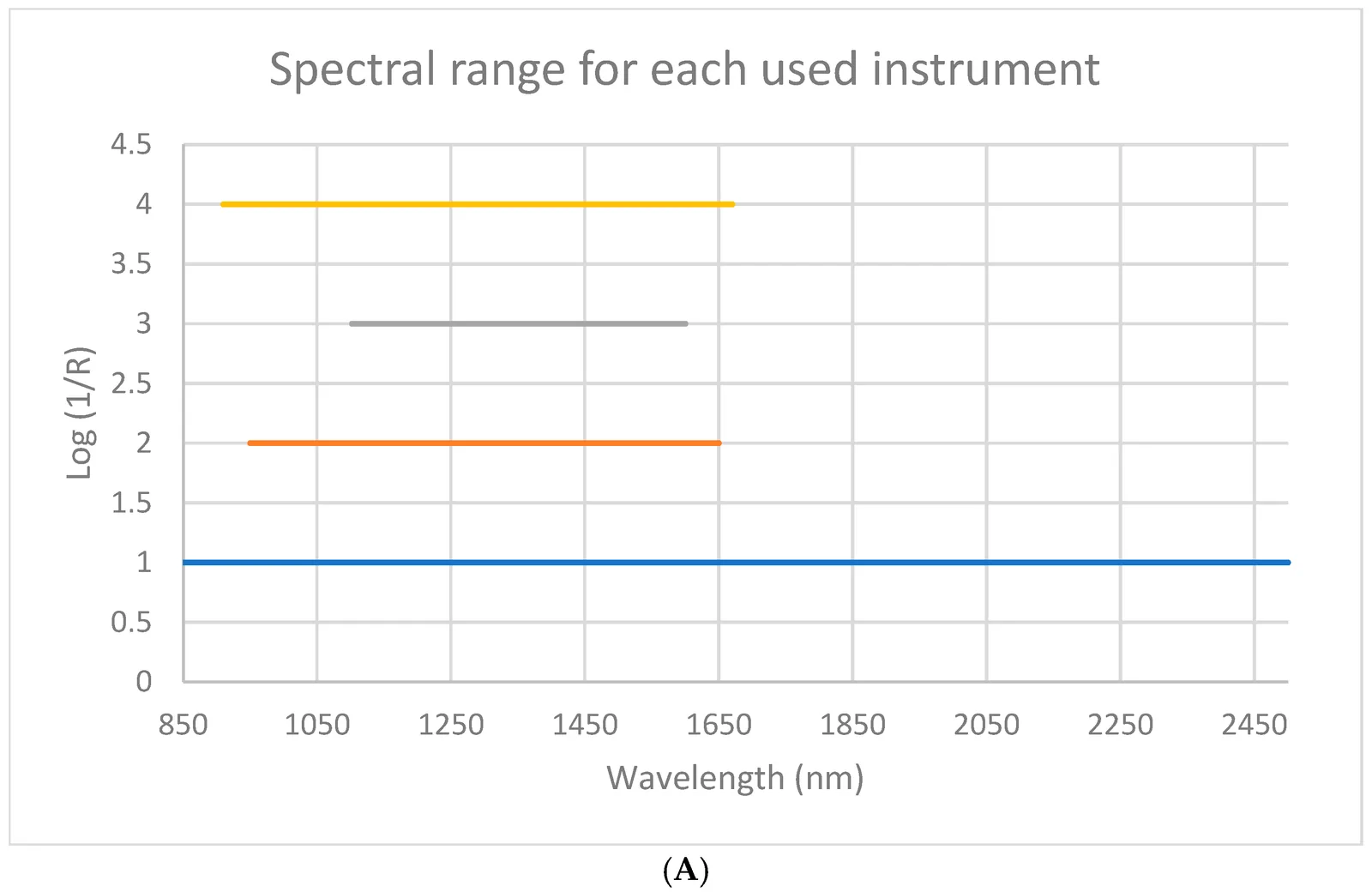

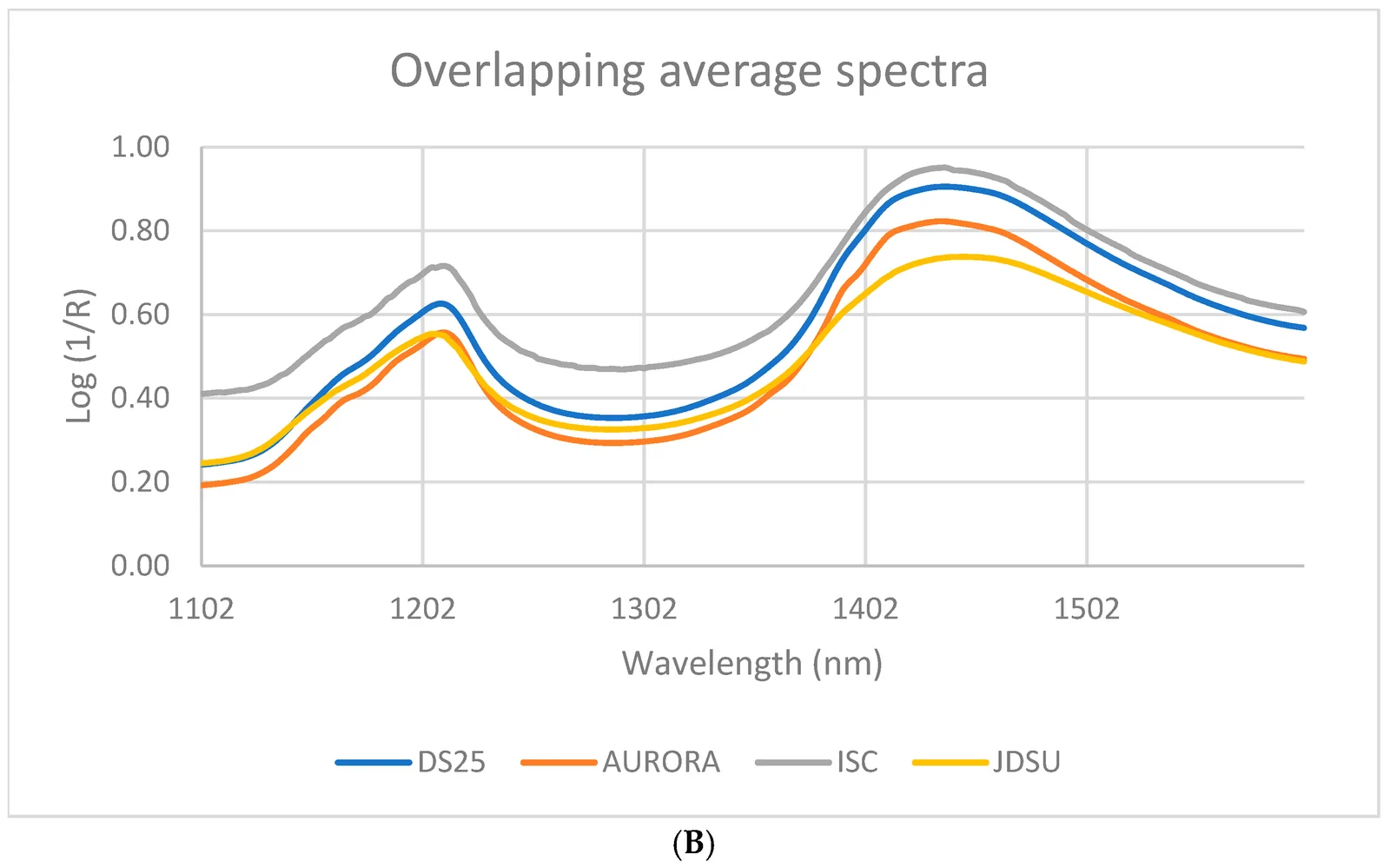
The spectral range extension for the four Near-infrared (NIR) systems (**A**) and the overlapping trimmed average spectra for the used NIR systems (**B**). NIR devices were: Foss DS 2500 (DS25), Aurora, ISC and JDSU.

**Figure 2:**
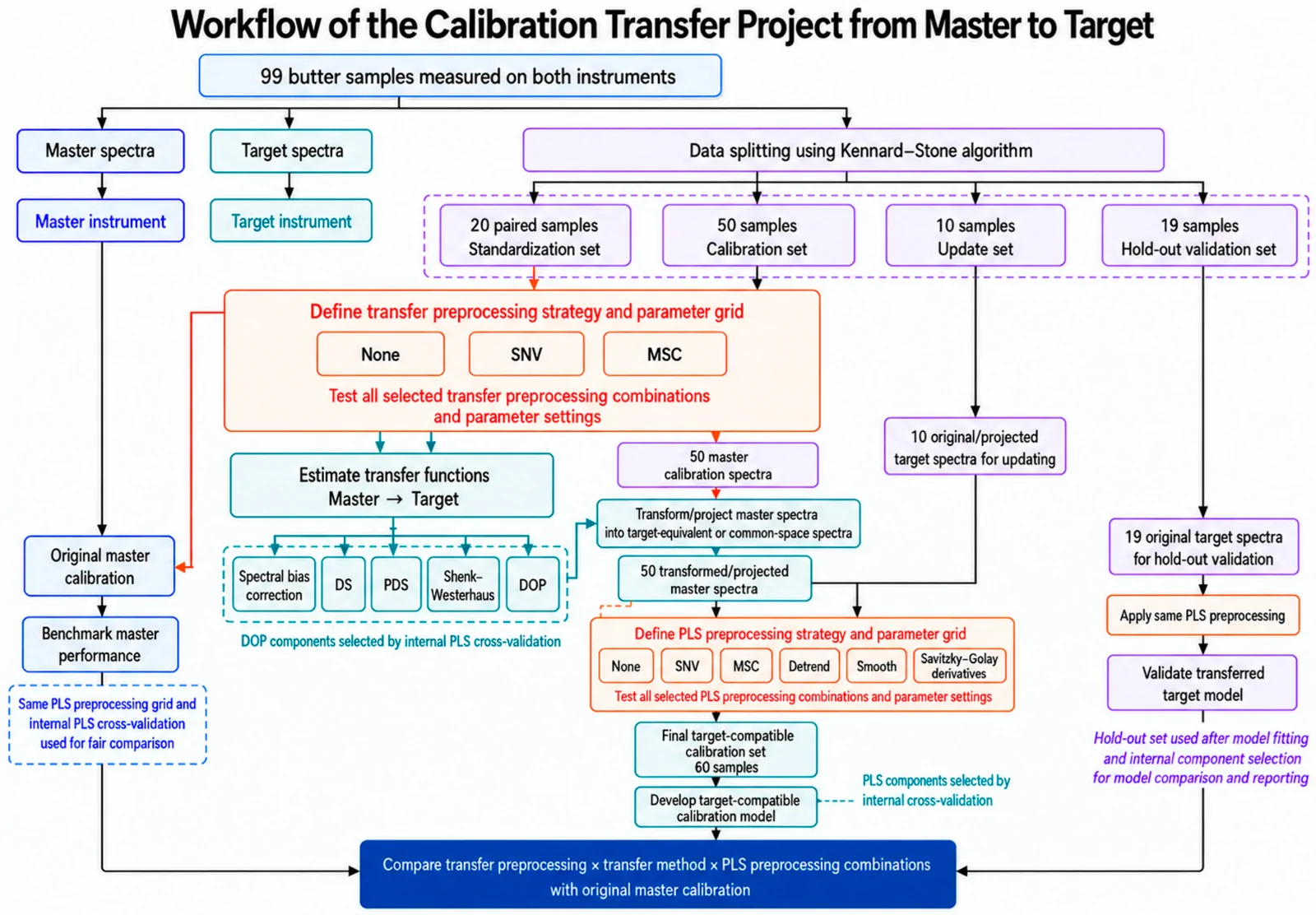
Workflow of calibration transfer from master to target instruments (targets), including spectral preprocessing optimization, standardization methods, internal partial least squares (PLS) cross-validation and hold-out validation. DS, direct standardization; PDS, piecewise direct standardization; DOP, dynamic orthogonal projection.

**Figure 3:**
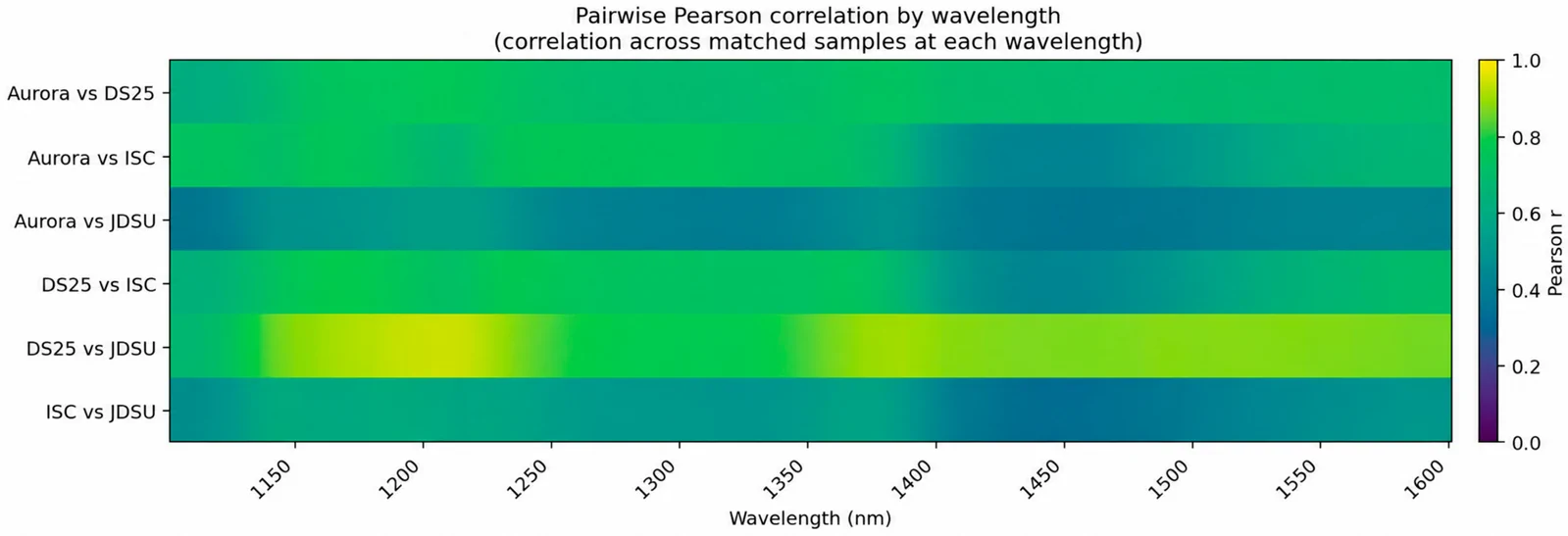
The pairwise Pearson coefficient of correlation (r) map drawn by wavelengths in common spectral range.

**Table 1:** Dataset used in the trial.

Subset	Samples	Spectra Used	Purpose
Standardization	20	Paired master and target spectra	Estimate the transfer relationship between master and target
Calibration	50	Master spectra	Transform/project master spectra into target-compatible spectra
Update	10	Original target spectra	Update final target-compatible calibration
Hold-out validation	19	Original target spectra	Evaluate fitted candidate target models and support comparative reporting

**Table 2:** Scenarios tested in the trial.

Scenario	Calibration Set	Hold-Out Validation Set	Purpose
DS25 benchmark	50 original DS25 + 10 original DS25	19 original DS25	Reference performance of the master instrument
Target transferred: SBC, DS, PDS, S-W	50 transformed master + 10 original target	19 original target	Evaluate spectral transfer from master to target
Target transferred: DOP	50 projected master + 10 projected target	19 projected target	Evaluate transfer after removal of instrumental difference directions

Abbreviations: DS, direct standardization; DS25, FOSS DS2500; DOP, dynamic orthogonal projection; PDS, piecewise direct standardization; SBC, spectral bias correction; S-W, Shenk-Westerhaus.

**Table 3:** Default parameter values used in the calibration transfer (CT) workflow and in the partial least squares (PLS) regression stage. Parameters are grouped by function; the values shown are the defaults set in the interactive script and are applied unless the operator provides alternative inputs at runtime.

Category	Parameter	Default Value	Description
Dataset split			
	N in standardization	20	Samples used to estimate transfer functions (paired master–target)
	N in calibration	50	Master spectra used to build the PLS calibration model
	N in update	10	Target spectra added to the calibration set after transfer
	N in validation	19	Hold-out validation samples; not used during model training or internal component selection
	Sample selection	Kennard–Stone	Samples assigned to each subset using Kennard–Stone selection in standardized spectral space
Cross-validation			
	N. of splits in cross-validation	5	Number of folds for k-fold cross-validation (shuffle = True)
	Random state	123	Random seed for reproducible fold assignment
PLS regression			
	Max PLS components	5	Maximum number of PLS latent variables tested during internal CV
	Scale	True	X and y variables are auto-scaled before PLS decomposition (scikit-learn PLSRegression default)
	Component selection	Min RMSECV	Optimal number of components chosen as the value minimizing RMSECV across all CV folds
Calibration transfer methods			
	SBC	—	Wavelength-wise additive shift: mean (target–master) over standardization samples; no tunable parameters
	DS ^a^–ridge regularisation (ridge alpha)	1 × 10^−6^	Ridge penalty applied during DS to stabilize the solution when standardization samples are fewer than wavelengths
	PDS ^a^–ridge regularisation (ridge alpha)	1 × 10^−6^	Same ridge penalty applied independently to each local window regression in PDS
	PDS–window sizes tested	3, 5, 7, 9, 11	Odd-numbered sliding window, window sizes tested (in wavelength points) evaluated by grid search
	S-W standardization	—	Wavelength-wise linear regression (slope + intercept) between master and target standardization spectra; no tunable parameters
	DOP ^b^–max components tested	5	Maximum number of PCA components of the difference matrix used to construct the DOP orthogonal projection; optimal number selected by internal PLS CV
Spectral preprocessing (transfer and PLS stages)			
	Default sequences tested (both stages)	See note ^c^	Transfer stage: none; SNV; MSC PLS stage: none; SNV; MSC; SNV + smooth; SNV + SG derivative + smooth; MSC + smooth; MSC + SG derivative + smooth
	SG smooth–window lengths	3, 5, 7	Odd-numbered window lengths (wavelength points) for Savitzky–Golay smoothing (deriv = 0)
	SG smooth–polynomial order	2	Polynomial degree for Savitzky–Golay smoothing; window_length must exceed polynomial order
	SG derivative–window lengths	3, 5, 7	Odd-numbered window lengths for Savitzky–Golay derivative
	SG derivative–polynomial order	2	Polynomial degree for Savitzky–Golay derivative computation
	SG derivative–derivative orders	1, 2	First and second derivatives evaluated in the grid search
	SG derivative–gap	3, 5, 7	Subsampling step applied after differentiation; gap = 1 retains all points; larger values reduce variable count
	Detrend	linear	Linear baseline removal applied independently to each spectrum (scipy.signal.detrend, type = “linear”)
	MSC reference	Mean spectrum	Reference spectrum for MSC computed as the column-wise mean of the calibration/update set; refitted inside each CV fold to prevent data leakage

^a^ Methods use ridge regression (Ridge, scikit-learn) with fit_intercept = True. ^b^ DOP = Dynamic Orthogonal Projection. Components of the difference matrix D = X_target_ − X_master_ are extracted by PCA (sklearn.decomposition.PCA). The projection matrix P is defined as I − V V^T^, where V contains the selected eigenvectors. ^c^ Transfer preprocessing and PLS preprocessing are configured independently. Transfer preprocessing is applied before estimating the master–target transfer function; PLS preprocessing is applied after transfer, immediately before PLS model fitting, and is refitted inside each cross-validation fold to prevent leakage. Abbreviations: CV, cross-validation; DS, direct standardization; DOP, dynamic orthogonal projection; MSC, multiplicative scatter correction; PDS, piecewise direct standardization; PLS, partial least squares; RMSECV, root mean square error of cross-validation; RMSEP, root mean square error of prediction; SG, Savitzky–Golay; SBC, spectral bias correction; SNV, standard normal variate; S-W, Shenk–Westerhaus.

**Table 4:** The average Pearson coefficient of correlation (r) between instruments.

	Aurora	DS25	ISC	JDSU
Aurora	-	0.94	0.91	0.91
DS25	0.94	-	0.92	0.98
ISC	0.91	0.92	-	0.91
JDSU	0.91	0.98	0.91	-

**Table 5:** Overall hold-out validation performance by target instrument of the three NIR portable devices (Aurora, JDSU and ISC); values are averages across the 32 response variables. The comparison with the master benchmark (Foss DS2500, DS25) uses the mean of the three DS25 benchmarks.

NIR Device	Mean RMSEP	Median RMSEP	Mean R^2^_pred_	Median R^2^_pred_	Mean RPD	Median RPD	Mean |Bias|	Mean RMSEP vs. Master (%)	*n* RMSEP Within ±10% of DS25	*n* RPD ≥ 2.0	*n* RPD ≥ 1.5
Aurora	0.29	0.19	0.62	0.62	1.96	1.67	0.03	−11.9	14	13	20
JDSU	0.29	0.21	0.57	0.56	1.89	1.56	0.05	−7.64	16	13	19
ISC	0.41	0.31	0.35	0.37	1.36	1.29	0.08	25.7	9	3	6

RMSEP, root mean square error of external prediction; RPD, ratio of performance to deviation; R^2^_pred_, coefficient of determination in external prediction. RMSEP vs. DS25 (%) was calculated as [(RMSEP_target}_ − RMSEP_master_)/RMSEP_master_] × 100. The column “*n* RMSEP within ±10% of DS25” indicates the number of response variables for which the transferred handheld model achieved an RMSEP within ±10% of the corresponding DS25 benchmark. RPD thresholds indicate the number of response variables that reach RPD ≥ 2.0 or ≥1.5.

**Table 6:** Distribution and mean performance of the selected transfer methods. The number of models indicates how often a transfer method was identified as the best-performing configuration on the hold-out validation set for a given response variable and instrument.

Instrument	Method	*n*	Mean RMSEP	Mean R^2^_pred_	Mean RPD	Mean |Bias|
Aurora	DOP	9	0.14	0.56	1.68	0.02
Aurora	SBC	8	0.19	0.73	2.36	0.03
Aurora	PDS	7	0.42	0.55	1.90	0.06
Aurora	S-W	5	0.18	0.60	1.94	0.02
Aurora	DS	3	0.85	0.66	1.91	0.03
JDSU	DOP	9	0.16	0.60	1.82	0.05
JDSU	PDS	9	0.38	0.40	1.65	0.04
JDSU	S-W	5	0.51	0.75	2.19	0.09
JDSU	DS	5	0.14	0.47	1.51	0.04
JDSU	SBC	4	0.30	0.80	2.73	0.05
ISC	PDS	11	0.36	0.29	1.35	0.04
ISC	DOP	7	0.54	0.43	1.46	0.14
ISC	S-W	7	0.57	0.45	1.40	0.13
ISC	SBC	5	0.20	0.26	1.23	0.02
ISC	DS	2	0.24	0.31	1.25	0.06

DOP, dynamic orthogonal projection; DS, direct standardization; PDS, piecewise direct standardization; RMSEP, root mean square error of external prediction; R^2^_pred_, coefficient of determination in external prediction SBC, spectral bias correction; S-W, Shenk–Westerhaus.

**Table 7:** Summary of the reduced top-five ensemble workflow for calibration transfer from DS25 to Aurora, ISC and JDSU portable NIR devices. The five configurations were selected using a composite ranking based on RMSEP, R^2^_pred_, absolute bias, RPD, and slope. Ensemble metrics (R^2^_pred,ens,_ RPD_ens_) were calculated from the averaged predictions of the five selected configurations. RMSEP change refers to the comparison between the ensemble prediction and the best single configuration retained within the selected top-five set. A total of 32 variables were evaluated.

	Variable				^a^ RMSEP			Mean R^2^_pred,ens_	Median R^2^_pred,ens_	Mean RPD_ens_
	R^2^ ≥ 0.70	R^2^ ≥ 0.50	^b^ Improved	Within ±5%	^c^ Worsening >5%	Meanchange (%)	Medianchange (%)			
Aurora	13	19	22	24	0	−3.32	−1.45	0.59	0.58	1.94
ISC	2	6	19	21	2	−2.79	−0.58	0.31	0.28	1.31
JDSU	13	18	17	25	1	−1.51	−0.15	0.54	0.63	1.83

^a^ RMSEP, root mean square error of external prediction; ^b^ Improved vs. best selected single model; ^c^ Variables with RMSEP worsening > 5%; R^2^_pred_, coefficient of determination in external prediction.

## Data Availability

The original data presented in the study are openly available at Research Data Unipd, the open-source repository of databases at the University of Padova at https://researchdata.cab.unipd.it/1890/ (accessed on 21 September 2026) with DOI: 10.25430/researchdata.cab.unipd.it.00001890.

## Supplementary Materials

The following supporting information can be downloaded at: https://www.mdpi.com/article/10.3390/s26196110/s1. Supplementary Tables S1 and S2: report, respectively, the best transferred handheld model for each response variable and the prediction-based consistency of calibration transfer across instruments. Supplementary File S1: contains the Python code for the calibration-transfer and PLS modeling workflow; Supplementary File S2: provides the Python code for the reduced top-five ensemble analysis; Supplementary File S3: contains the Python code used to generate the official Kennard–Stone sample split from the DS25 master spectra; Supplementary File S4: provides the corresponding fixed allocation of the 99 samples to the standardization, calibration, update, and hold-out validation subsets.

## Abbreviations

The following abbreviations are used in this manuscript:

CLA	Conjugated linoleic acid
CT	Calibration transfer
CV	Cross-validation
DOP	Dynamic orthogonal projection
DS	Direct standardization
DS25	FOSS DS2500 benchtop NIR instrument
FA	Fatty acids
FAME	Fatty acid methyl esters
GC	Gas chromatography
ISC	Integrated Spectronics handheld NIR spectrometer
JDSU	JDS Uniphase handheld NIR spectrometer
MAE	Mean absolute error
MSC	Multiplicative scatter correction
MUFA	Monounsaturated fatty acids
NIR	Near-infrared
PCA	Principal component analysis
PDS	Piecewise direct standardization
PLS	Partial least squares
PUFA	Polyunsaturated fatty acids
R^2^_pred_	Coefficient of determination in hold-out prediction
R^2^_pred,ens_	Coefficient of determination in hold-out prediction for the ensemble
RMSE	Root mean square error
RMSEC	Root mean square error of calibration
RMSE_consistency_	Root mean square error of prediction-consistency
RMSECV	Root mean square error of cross-validation
RMSEP	Root mean square error of prediction
RMSEP_ens_	Root mean square error of prediction for the ensemble prediction
RPD	Ratio of performance to deviation
RPD_ens_	Ratio of performance to deviation for the ensemble prediction
SBC	Spectral bias correction
SEP	Standard error of prediction after bias correction
SFA	Saturated fatty acids
SG	Savitzky–Golay
SNV	Standard normal variate
SRD	Sum of ranking differences
S-W	Shenk–Westerhaus
TMR	Total mixed ration
