# Supplementary material for: From Benchtop to Handheld Systems: Calibration Transfer for On-Site NIR System Butter Quality Monitoring
Source: Sensors (Basel). 2026 Sep 26;26(19):6110. doi: 10.3390/s26196110 (PMC13667566; doi:10.3390/s26196110)
Supplement: Supplementary file 1 [file sensors-26-06110-s001.zip › Sensors 4504687 - supplementary tables S1 and S2.pdf]

Table S1. Best transferred target model for each response variable. The best target model was defined as the model with the lowest RMSEP among Aurora, JDSU, and ISC for each response variable. The master benchmark is the FOSS DS2500 (DS25) benchmark results.

Response	Best target	Method	RMSEP	R^2_{pred}	RPD	Bias	Master RMSEP	Master R^2_{pred}	Master RPD	RMSEP vs master (%)
Chemical										
Moisture	Aurora	PDS	0.51	0.90	3.31	–0.06	0.59	0.87	2.88	–12.8
Fat	JDSU	PDS	0.52	0.90	3.21	0.03	0.62	0.86	2.71	–15.5
Fatty acid profile										
C4:0	Aurora	PDS	0.33	0.27	1.20	0.00	0.36	0.15	1.11	–7.56
C6:0	JDSU	SBC	0.11	0.54	1.52	0.00	0.15	0.10	1.09	–28.5
C8:0	JDSU	S-W	0.07	0.56	1.54	0.01	0.08	0.36	1.28	–16.8
C10:0	JDSU	SBC	0.10	0.87	2.89	0.05	0.11	0.84	2.58	–10.8
C12:0	JDSU	DOP	0.18	0.75	2.04	0.08	0.19	0.72	1.93	–5.20
C14:0	Aurora	SBC	0.33	0.84	2.60	–0.02	0.37	0.81	2.37	–8.82
C14:1	Aurora	S-W	0.04	0.64	1.71	0.01	0.05	0.49	1.44	–16.0
C15:0	Aurora	DOP	0.07	0.28	1.21	–0.01	0.08	0.13	1.10	–8.94
C16:0	Aurora	DS	0.96	0.69	1.85	–0.03	0.87	0.75	2.05	10.6
C16:1	Aurora	DOP	0.09	0.36	1.29	–0.03	0.11	0.03	1.04	–19.0
C17:0	Aurora	PDS	0.03	0.58	1.58	–0.01	0.04	0.20	1.15	–27.1
C17:1	JDSU	PDS	0.02	0.41	1.34	–0.01	0.03	0.26	1.20	–10.4
C18:0	Aurora	DS	0.37	0.47	1.41	0.06	0.39	0.42	1.35	–4.57
C18:1	JDSU	SBC	0.46	0.89	3.05	0.08	0.63	0.78	2.19	–28.2
C18:1 *t*-11	Aurora	SBC	0.20	0.91	3.37	0.00	0.22	0.88	3.02	–10.4
C18:2 *n*-6	JDSU	DOP	0.23	0.57	1.57	–0.02	0.35	0.01	1.03	–34.2
C18:2 *c*-9, *t*-11	Aurora	SBC	0.07	0.86	2.79	0.01	0.09	0.82	2.43	–12.8
C18:3 *n*-6	Aurora	DOP	0.08	0.55	1.53	–0.01	0.08	0.48	1.43	–6.57
C18:3 *n*-3	Aurora	DOP	0.09	0.56	1.54	–0.02	0.10	0.47	1.41	–8.36
C20:0	ISC	SBC	0.03	0.26	1.19	0.00	0.04	0.13	1.10	–7.73
C20:4 *n*-6	JDSU	PDS	0.04	0.19	1.14	0.00	0.04	0.05	1.06	–7.46
Calculated FA										
Σ SFA	JDSU	S-W	1.11	0.85	2.69	0.10	1.01	0.88	2.95	9.74
Σ MUFA	JDSU	SBC	0.55	0.91	3.48	–0.07	0.69	0.87	2.80	–19.4
Σ PUFA	Aurora	DOP	0.27	0.83	2.52	–0.04	0.40	0.63	1.69	–33.1
Σ PUFA *n*-6	Aurora	DOP	0.23	0.82	2.41	0.00	0.35	0.59	1.60	–33.7
Σ PUFA *n*-3	Aurora	SBC	0.09	0.60	1.63	–0.02	0.10	0.54	1.52	–6.70
Σ SCFA	Aurora	PDS	0.52	0.50	1.45	0.12	0.63	0.29	1.22	–16.4
Σ CLA	Aurora	S-W	0.07	0.87	2.80	–0.02	0.09	0.82	2.43	–13.3
Σ *n*-6	JDSU	DOP	0.23	0.69	1.83	–0.06	0.38	0.13	1.10	–39.9
n-6 over *n*-3	ISC	PDS	0.85	0.33	1.25	0.03	0.96	0.14	1.11	–11.6

FA, fatty acids; SFA, saturated FA; MUFA, monounsaturated FA; PUFA, polyunsaturated FA; SCFA, short-chain FA; CLA, conjugated linoleic acid; DOP, Dynamic Orthogonal Projection; DS, Direct Standardization; PDS, Piecewise Direct Standardization; RMSEP, root mean square error of external prediction; R^2_{pred}, coefficient of determination in external prediction. S-W, Shenk-Westerhaus; SBC, Spectral bias correction.

Table S2. Prediction-based consistency of calibration transfer across instruments for chemical traits and fatty acid profile.

Item	RMSEP				$RMSE_{consistency}$			Bias vs DS25			MCS
	DS25	Aurora	JDSU	ISC	Aurora	JDSU	ISC	Aurora	JDSU	ISC	
Chemical											
Moisture	0.59	0.51	0.53	0.73	0.17	0.43	0.66	0.07	0.18	0.28	Aurora
Fat	0.62	0.53	0.52	0.74	0.19	0.50	0.65	−0.09	−0.16	−0.35	Aurora
Fatty acid profile											
C4:0	0.36	0.33	0.34	0.35	0.04	0.03	0.10	−0.03	−0.04	−0.08	JDSU
C6:0	0.15	0.12	0.11	0.12	0.06	0.06	0.07	−0.07	−0.05	−0.04	JDSU
C8:0	0.08	0.08	0.07	0.08	0.05	0.02	0.02	−0.06	−0.03	−0.03	ISC
C10:0	0.11	0.10	0.10	0.19	0.10	0.04	0.14	−0.04	−0.01	−0.05	JDSU
C12:0	0.19	0.18	0.18	0.27	0.10	0.05	0.17	−0.06	−0.03	−0.08	JDSU
C14:0	0.37	0.34	0.40	0.43	0.25	0.28	0.33	−0.10	0.04	0.10	Aurora
C14:1	0.05	0.04	0.05	0.07	0.02	0.04	0.04	−0.01	−0.03	−0.02	Aurora
C15:0	0.08	0.07	0.07	0.08	0.03	0.01	0.03	−0.04	−0.03	−0.04	JDSU
C16:0	0.87	0.96	1.00	1.31	0.64	0.42	0.86	−0.10	0.19	0.21	JDSU
C16:1	0.11	0.09	0.09	0.09	0.05	0.04	0.05	−0.08	−0.03	−0.04	JDSU
C17:0	0.04	0.03	0.04	0.04	0.02	0.01	0.02	0.00	0.00	0.01	JDSU
C17:1	0.03	0.02	0.02	0.02	0.01	0.01	0.01	0.00	−0.01	−0.01	Aurora
C18:0	0.39	0.37	0.43	0.45	0.19	0.22	0.31	0.11	−0.01	0.18	Aurora
C18:1	0.63	0.54	0.46	0.97	0.31	0.34	0.92	−0.15	−0.13	−0.42	Aurora
C18:1 *t*-11	0.22	0.20	0.28	0.50	0.24	0.18	0.40	−0.02	−0.08	−0.18	JDSU
C18:2 *n*-6	0.35	0.24	0.23	0.35	0.19	0.10	0.25	0.17	0.15	0.21	JDSU
C18:2 *c*-9, *t*-11	0.09	0.08	0.09	0.15	0.07	0.06	0.12	0.00	−0.03	−0.02	JDSU
C18:3 *n*-6	0.08	0.08	0.08	0.11	0.06	0.04	0.05	0.01	0.02	0.01	JDSU
C18:3 *n*-3	0.10	0.10	0.11	0.14	0.02	0.04	0.07	0.00	0.00	−0.02	Aurora
C20:0	0.04	0.03	0.04	0.03	0.01	0.01	0.01	0.01	0.00	0.01	JDSU
C20:4 *n*-6	0.04	0.04	0.04	0.04	0.01	0.01	0.01	0.00	0.01	0.00	ISC
Calculated FA											
Σ SFA	1.01	1.21	1.11	1.69	0.58	0.87	1.39	0.12	0.23	0.56	Aurora
Σ MUFA	0.69	0.72	0.55	1.20	0.39	0.38	0.74	−0.13	−0.27	0.08	JDSU
Σ PUFA	0.40	0.27	0.28	0.56	0.22	0.17	0.37	0.21	0.14	0.09	JDSU
Σ PUFA *n*-6	0.35	0.23	0.24	0.40	0.17	0.12	0.26	0.21	0.13	0.10	JDSU
Σ PUFA *n*-3	0.10	0.09	0.10	0.14	0.05	0.07	0.08	0.00	0.04	0.00	Aurora
Σ SCFA	0.63	0.52	0.56	0.61	0.18	0.18	0.29	0.02	−0.04	−0.09	JDSU
Σ CLA	0.09	0.07	0.08	0.14	0.06	0.04	0.11	−0.03	−0.03	−0.02	JDSU
Σ *n*-6	0.38	0.24	0.23	0.34	0.19	0.11	0.20	0.19	0.14	0.17	JDSU
n-6 over *n*-3	0.96	0.87	0.93	0.85	0.52	0.22	0.32	−0.25	−0.28	−0.09	JDSU

FA, fatty acids; SFA, saturated FA; MUFA, monounsaturated FA; PUFA, polyunsaturated FA; SCFA, short-chain FA; CLA, conjugated linoleic acid; MCS, most consistent target. RMSEP was calculated against reference analytical values and describes prediction accuracy. $RMSE_{consistency}$ was calculated against the median prediction across instruments for the same validation samples and describes instrument-to-instrument agreement. Bias vs master FOSS DS2500 (DS25) was calculated as target prediction minus DS25 prediction; RMSEP, root mean square error of external prediction.
