# Supplementary material for: From Benchtop to Handheld Systems: Calibration Transfer for On-Site NIR System Butter Quality Monitoring
Source: Sensors (Basel). 2026 Sep 26;26(19):6110. doi: 10.3390/s26196110 (PMC13667566; doi:10.3390/s26196110)
Supplement: Supplementary file 1 [file sensors-26-06110-s001.zip › sensors-4504687-supplementary S1.pdf]

Supplementary File S1: Python code for the calibration-transfer and PLS modelling workflow

```
# -*- coding: utf-8 -*-
"""

#
======================================================================
====
# READING GUIDE
#
======================================================================
====
# This script may look long because it performs the full calibration-
transfer
# workflow automatically. The main idea is simple:
#
# 1. Read three files:
#       - one file with spectra from the master instrument;
#       - one file with spectra from the slave instrument;
#       - one file with the chemical reference values.
#
# 2. Match the rows of the three files using the sample ID column.
#    This ensures that the same butter sample is compared across all
files.
#
# 3. Split samples into four groups:
#       - standardization set: used to learn how master and slave differ;
#       - calibration set: master spectra that will be transferred to
slave space;
#       - update set: fresh slave spectra added to adapt the calibration;
#       - validation set: samples kept separate from preprocessing
fitting, transfer-function estimation, calibration/update, and internal
component selection, and subsequently used for hold-out model evaluation
and comparison.
#
# 4. Test the selected preprocessing and calibration-transfer methods.
#    Transfer preprocessing and PLS preprocessing are separated:
#       - transfer preprocessing is used before estimating master ->
slave transfer;
#       - PLS preprocessing is used after transfer, before building the
PLS model.
#
# 5. Build PLS models and test them on hold-out validation samples.
#
# 6. Save all results and all author-defined parameters in one Excel
workbook.
#
# Practical note:
#     Usually, you should edit only the variables in Section 2, such as
DATA_DIR,
#     MASTER_FILE, SLAVE_FILE, CHEMICAL_FILE, ID_COL, and the split
sizes.
#
======================================================================
====

Calibration transfer from master to slave NIR spectra for butter samples.
```

```
Interactive script for Spyder 6.1.0 / Anaconda.

UPDATED WORKFLOW
----------------
This version separates two different preprocessing stages:

1. Transfer preprocessing
   - Applied before estimating/applying the instrument standardization
method.
   - Used to estimate master -> slave transfer functions on the
standardization set.
   - Can be set to "none" if the user wants no preprocessing during
transfer.

2. PLS preprocessing
   - Applied after transfer/projection, immediately before PLS modelling.
   - Fitted only on the final calibration/update dataset.
   - Applied to the hold-out validation set using parameters fitted on
calibration data.
   - Can be different from transfer preprocessing.

Example:
    Transfer preprocessing = none
    PLS preprocessing = snv + sg_derivative

Experimental design
-------------------
Data split is performed before any preprocessing or modelling:
    - 20 standardization samples
    - 50 calibration samples
    - 10 update samples
    - 19 hold-out validation samples

For slave-compatible models:
    - transfer functions are estimated on the 20 paired standardization
samples;
    - 50 master calibration spectra are transformed/projected toward the
slave space;
    - 10 original/projected slave update spectra are added;
    - PLS preprocessing is fitted on this 60-sample dataset;
    - PLS components are selected by internal cross-validation;
    - Candidate models are evaluated on the hold-out slave validation set
only after model fitting and internal PLS/DOP component selection have
been completed; hold-out validation metrics are subsequently used for
comparative ranking and reporting.

For DOP:
    - DOP components are selected by internal PLS cross-validation;
    - the hold-out validation set is not used to select DOP components.

"""

#
==========================================================================
====
# 1. Import libraries
#
==========================================================================
====
```

```
# Libraries are external Python packages used by the script.
# - os handles file paths.
# - json stores parameter choices as readable text in Excel.
# - itertools creates all combinations of preprocessing parameters.
# - numpy and pandas manage numerical matrices and tables.
# - scipy performs spectral smoothing, derivatives, and detrending.
# - scikit-learn performs PCA, PLS regression, cross-validation, and
metrics.

import os
import json
import pickle
import itertools
import warnings

import numpy as np
import pandas as pd

from scipy.signal import savgol_filter
from scipy import signal

from sklearn.cross_decomposition import PLSRegression
from sklearn.model_selection import KFold
from sklearn.metrics import r2_score, mean_absolute_error
from sklearn.preprocessing import StandardScaler
from sklearn.decomposition import PCA
from sklearn.linear_model import LinearRegression, Ridge

warnings.filterwarnings("ignore")


#
==========================================================================
====
# 2. Fixed file and analysis settings
#
==========================================================================
====
# This is the main section to edit before running the script.
# It defines where the input files are located, how sample IDs are named,
# which chemical parameters are modelled, and how many samples go into
each set.

# Main folder on the local PC.
# The script will look here for the master, slave, and chemical files.
# If this folder is not found, the script will automatically use the
folder
# where this .py file is saved.
DATA_DIR =r"C:\..."

# Input file names.
# MASTER_FILE is the calibrated/reference instrument.
# SLAVE_FILE is the instrument to which the calibration is transferred.
# CHEMICAL_FILE contains the laboratory reference values used as y
variables.
# Edit these names if your files have different names.
MASTER_FILE = "DS2500.csv"
SLAVE_FILE = "Aurora.csv"
CHEMICAL_FILE = "chemical_data.xlsx"
```

```
# Column containing the sample identifier.
# This column must exist in all three files.
# The script uses it to make sure the same sample is aligned across
files.
ID_COL = "ID"

# Chemical parameters to model.
# Each name listed here must match one column in the chemical file.
# The script will build one model for each parameter in this list.
RESPONSE_COLUMNS = [
    "Moisture", "FatD", "n6",
    "SFA", "MUFA", "Short", "PUFAn6", "PUFAn3",
    "PUFA", "CLA", "n6_n3",  "C4_0",  "C6_0",  "C8_0", "C10_0", "C12_0",
"C14_0", "C14_1n9",
      "C15_0", "C16_0", "C16_1n9", "C17_0", "C17_1n9", "C18_0",
      "C18_1n9ct", "C18_1n11trans-Vacc.", "C18_2n6c", "C18_3n6",
      "C18_3n3", "C18_2c9t11", "C20_0", "C20_4n6"
]

# Fixed data split sizes.
# The sum of these four numbers must equal the number of samples that are
# common to the master, slave, and chemical files.
# Example for 99 samples: 20 + 50 + 10 + 19 = 99.
# Example for 101 samples: 20 + 50 + 11 + 20 = 101.
N_STANDARDIZATION = 20
N_CALIBRATION = 50
N_UPDATE = 10
N_VALIDATION = 19

# Internal cross-validation settings.
# Cross-validation is used only inside the calibration set to choose
model
# complexity, such as the number of PLS components.
# The hold-out validation set is not used for these choices.
N_SPLITS_CV = 5
RANDOM_STATE = 123

# Ridge regularization used for DS and PDS.
# This small value stabilizes matrix calculations when many wavelengths
are
# available but relatively few standardization samples are used.
RIDGE_ALPHA = 1e-6

# Output file names.
# The Excel workbook contains detailed results and author-defined
parameters.
# The CSV file contains the full result table.
# The split CSV records which sample belongs to each dataset.
OUTPUT_EXCEL =
"calibration_transfer_results_separate_transfer_and_pls_preprocessing.xls
x"
OUTPUT_CSV =
"calibration_transfer_results_separate_transfer_and_pls_preprocessing.csv
"
# Full validation predictions can become too large for one Excel sheet.
# They are therefore saved as a separate CSV file.
OUTPUT_PREDICTIONS_CSV = "validation_predictions_full.csv"
# Python workspace file containing the main result objects for future
reuse in Spyder/Python.
OUTPUT_WORKSPACE = "calibration_transfer_workspace.pkl"
```

```
# Official Kennard-Stone split file created by the separate one-time
script.
# The main calibration-transfer script will import this file instead of
# recalculating Kennard-Stone at every run. This ensures that the same
samples
# always remain in the same subsets.
KS_SPLIT_FILE = "kennard_stone_split_official.csv"

# If True, this script requires KS_SPLIT_FILE to exist and imports it.
# If False, the script recalculates Kennard-Stone and saves OUTPUT_SPLIT.
# For reproducible analyses and publication, keep this as True after
creating
# the official split once with create_kennard_stone_split_once.py.
USE_EXISTING_KS_SPLIT = True

# Backup output name used only if USE_EXISTING_KS_SPLIT = False.
OUTPUT_SPLIT = "kennard_stone_split.csv"


#
==========================================================================
====
# 3. Interactive input utilities
#
==========================================================================
====
# These small helper functions ask the user questions in the Spyder
console.
# They also check that the answers make sense, for example that window
sizes
# are odd numbers when required by Savitzky-Golay filtering.

def ask_string(prompt, default_value):
    """Ask the user for a string input; return the default if ENTER is
pressed."""
    value = input(f"{prompt} [default: {default_value}]: ").strip()
    return default_value if value == "" else value


def ask_int(prompt, default_value, min_value=None, max_value=None):
    """Ask the user for an integer input with optional bounds."""
    while True:
        value = input(f"{prompt} [default: {default_value}]: ").strip()
        if value == "":
            return default_value
        try:
            value = int(value)
        except ValueError:
            print("Please enter an integer value.")
            continue
        if min_value is not None and value < min_value:
            print(f"Value must be >= {min_value}.")
            continue
        if max_value is not None and value > max_value:
            print(f"Value must be <= {max_value}.")
            continue
        return value
```

```
def ask_int_list(prompt, default_values, only_odd=False, min_value=None):
    """Ask the user for a comma-separated list of integers."""
    default_text = ",".join([str(x) for x in default_values])
    while True:
        value = input(f"{prompt} [default: {default_text}]: ").strip()
        if value == "":
            values = list(default_values)
        else:
            try:
                values = [int(x.strip()) for x in value.split(",") if
x.strip() != ""]
            except ValueError:
                print("Please enter comma-separated integer values, e.g.
5,7,9.")
                continue
        if len(values) == 0:
            print("At least one value is required.")
            continue
        if min_value is not None and any(x < min_value for x in values):
            print(f"All values must be >= {min_value}.")
            continue
        if only_odd and any(x % 2 == 0 for x in values):
            print("All window values must be odd.")
            continue
        return values


#
==========================================================================
====
# 4. Interactive configuration of transfer and PLS preprocessing grids
#
==========================================================================
====
# This part asks which spectral preprocessing strategies should be
tested.
# Transfer preprocessing and PLS preprocessing are deliberately separate:
# - transfer preprocessing affects the estimation of the master -> slave
relation;
# - PLS preprocessing affects the final prediction model after transfer.

def ask_preprocessing_sequences(stage_name, default_sequences):
    """
    Ask which preprocessing sequences should be tested for a specific
stage.

    stage_name:
        Used only for console messages, e.g. "TRANSFER" or "PLS".

    The order is controlled by the user using '+'. Different sequences
are
    separated by commas.
    """
    allowed_steps = ["none", "snv", "msc", "detrend", "smooth",
"sg_derivative"]

    print("\n" + "-" * 80)
    print(f"{stage_name} preprocessing sequences")
    print("-" * 80)
    print("Available preprocessing steps:")
```

```
    for step in allowed_steps:
        print(f"  - {step}")

    print("\nWrite the preprocessing sequences to test.")
    print("Use '+' to define the order within a sequence.")
    print("Use ',' to separate different sequences.")
    print("Example: none, snv, msc, snv+smooth, snv+sg_derivative")

    user_input = ask_string(f"\n{stage_name} preprocessing sequences to
test", default_sequences)

    sequences = []
    for item in user_input.split(","):
        item = item.strip().lower()
        if item == "":
            continue
        steps = tuple([x.strip().lower() for x in item.split("+")])
        for step in steps:
            if step not in allowed_steps:
                raise ValueError(f"Unknown preprocessing step: {step}.
Allowed: {allowed_steps}")
        if "none" in steps and len(steps) > 1:
            raise ValueError("'none' must be used alone.")
        sequences.append(steps)

    return sequences if len(sequences) > 0 else [("none",)]


def ask_preprocessing_parameter_grid(preprocessing_sequences,
stage_name):
    """Ask grid values only for preprocessing steps selected for a given
stage."""
    selected_steps = set()
    for sequence in preprocessing_sequences:
        for step in sequence:
            selected_steps.add(step)

    grid = {
        "none": [{}],
        "snv": [{}],
        "msc": [{}],
        "detrend": [{"type": "linear"}],
        "smooth": [],
        "sg_derivative": []
    }

    if "smooth" in selected_steps:
        print(f"\n{stage_name}: Savitzky-Golay smoothing parameter grid")
        smooth_windows = ask_int_list(
            f"{stage_name} smoothing window lengths, odd numbers",
            default_values=[3, 5, 7],
            only_odd=True,
            min_value=3
        )
        smooth_polyorders = ask_int_list(
            f"{stage_name} smoothing polynomial orders",
            default_values=[2],
            min_value=1
        )
        for window_length in smooth_windows:
```

```
            for polyorder in smooth_polyorders:
                if window_length > polyorder:
                    grid["smooth"].append({
                        "window_length": window_length,
                        "polyorder": polyorder
                    })
        if len(grid["smooth"]) == 0:
            raise ValueError(f"No valid {stage_name} smoothing parameter
combination was generated.")

    if "sg_derivative" in selected_steps:
        print(f"\n{stage_name}: Savitzky-Golay derivative parameter
grid")
        derivative_windows = ask_int_list(
            f"{stage_name} derivative window lengths, odd numbers",
            default_values=[3, 5, 7],
            only_odd=True,
            min_value=3
        )
        derivative_polyorders = ask_int_list(
            f"{stage_name} derivative polynomial orders",
            default_values=[2],
            min_value=1
        )
        derivative_orders = ask_int_list(
            f"{stage_name} derivative orders",
            default_values=[1, 2],
            min_value=1
        )
        derivative_gaps = ask_int_list(
            f"{stage_name} derivative gaps",
            default_values=[1, 3, 5, 7],
            min_value=1
        )
        for window_length in derivative_windows:
            for polyorder in derivative_polyorders:
                for deriv in derivative_orders:
                    for gap in derivative_gaps:
                        if window_length > polyorder:
                            grid["sg_derivative"].append({
                                "window_length": window_length,
                                "polyorder": polyorder,
                                "deriv": deriv,
                                "gap": gap
                            })
        if len(grid["sg_derivative"]) == 0:
            raise ValueError(f"No valid {stage_name} derivative parameter
combination was generated.")

    return grid


def expand_preprocessing_grid(preprocessing_sequences,
preprocessing_parameter_grid):
    """Expand selected preprocessing sequences into all parameter
combinations."""
    configs = []
    for sequence in preprocessing_sequences:
        param_lists = [preprocessing_parameter_grid[step] for step in
sequence]
```

```
        for params_combination in itertools.product(*param_lists):
            label_parts = []
            for step, params in zip(sequence, params_combination):
                if len(params) == 0:
                    label_parts.append(step)
                else:
                    param_text = "_".join([f"{k}{v}" for k, v in
params.items()])
                    label_parts.append(f"{step}({param_text})")
            configs.append({
                "sequence": sequence,
                "params": params_combination,
                "label": " + ".join(label_parts)
            })
    return configs


def ask_model_parameter_grid():
    """Ask model and transfer parameter grid values."""
    print("\nModel parameter grid")
    max_pls_components = ask_int(
        "Maximum number of PLS components to test",
        default_value=5,
        min_value=1
    )
    max_dop_components = ask_int(
        "Maximum number of DOP components to test",
        default_value=5,
        min_value=1
    )
    pds_windows = ask_int_list(
        "PDS window sizes, odd numbers",
        default_values=[3, 5, 7, 9, 11],
        only_odd=True,
        min_value=3
    )
    return {
        "max_pls_components": max_pls_components,
        "max_dop_components": max_dop_components,
        "pds_windows": pds_windows
    }


#
=========================================================================
====
# 5. File reading and sample alignment
#
=========================================================================
====
# This section reads the input files and checks that the samples match.
# The most important point is that the same ID must refer to the same
butter
# sample in the master spectra, slave spectra, and chemical data.

def resolve_data_dir(data_dir):
    """Use DATA_DIR if it exists; otherwise use the folder containing
this script."""
    if os.path.isdir(data_dir):
        return data_dir
```

```
    script_dir = os.path.dirname(os.path.abspath(__file__))
    print("\nWARNING: DATA_DIR was not found.")
    print(f"Using the script folder instead: {script_dir}")
    return script_dir


def clean_column_names(df):
    """
    Clean column names after file import.

    This removes leading/trailing spaces and hidden UTF-8 BOM characters
    that can make a column visually appear as "ID" while pandas reads it
    as something different, e.g. "\ufeffID" or " ID ".
    """
    df = df.copy()
    df.columns = (
        df.columns
        .astype(str)
        .str.replace("\ufeff", "", regex=False)
        .str.strip()
    )
    return df


def read_table(filepath):
    """
    Read CSV or Excel files and clean column names.

    CSV files are read with automatic separator detection. This is safer
than
    forcing a comma or semicolon because spectral files may be exported
with
    different delimiters depending on the software/regional settings.
    """
    if not os.path.exists(filepath):
        raise FileNotFoundError(
            f"Input file not found:\n{filepath}\n"
            "Please check DATA_DIR and the input file names."
        )

    ext = os.path.splitext(filepath)[1].lower()

    if ext == ".csv":
        # sep=None with engine='python' lets pandas infer comma,
semicolon, tab, etc.
        df = pd.read_csv(filepath, sep=None, engine="python",
encoding="utf-8-sig")
    elif ext in [".xlsx", ".xls"]:
        df = pd.read_excel(filepath)
    else:
        raise ValueError(f"Unsupported file format: {ext}")

    return clean_column_names(df)


def get_spectral_columns(df, id_col="ID"):
    """Identify wavelength columns using numeric column names."""
    spectral_cols = []
    for col in df.columns:
        if col == id_col:
```

```
            continue
        try:
            float(col)
            spectral_cols.append(col)
        except ValueError:
            continue
    return sorted(spectral_cols, key=lambda x: float(x))


def align_master_slave_chemical(master_df, slave_df, chem_df,
id_col="ID"):
    """Align master spectra, slave spectra and chemical data by sample
ID."""
    master_df = clean_column_names(master_df.copy())
    slave_df = clean_column_names(slave_df.copy())
    chem_df = clean_column_names(chem_df.copy())

    # Provide an explicit and informative error if the ID column is
missing.
    # This is more useful than the default pandas KeyError.
    for file_label, df in [
        ("master file", master_df),
        ("slave file", slave_df),
        ("chemical file", chem_df)
    ]:
        if id_col not in df.columns:
            raise KeyError(
                f"Column '{id_col}' was not found in the {file_label}.\n"
                f"Available columns are: {df.columns.tolist()[:40]}\n"
                "Please verify ID_COL or rename the sample identifier
column."
            )

    master_df[id_col] = master_df[id_col].astype(str).str.strip()
    slave_df[id_col] = slave_df[id_col].astype(str).str.strip()
    chem_df[id_col] = chem_df[id_col].astype(str).str.strip()

    common_ids = (
        set(master_df[id_col])
        .intersection(set(slave_df[id_col]))
        .intersection(set(chem_df[id_col]))
    )
    common_ids = sorted(list(common_ids))

    if len(common_ids) == 0:
        raise ValueError("No common sample IDs were found.")

    master_df =
master_df[master_df[id_col].isin(common_ids)].sort_values(id_col).reset_i
ndex(drop=True)
    slave_df =
slave_df[slave_df[id_col].isin(common_ids)].sort_values(id_col).reset_ind
ex(drop=True)
    chem_df =
chem_df[chem_df[id_col].isin(common_ids)].sort_values(id_col).reset_index
(drop=True)

    if not np.all(master_df[id_col].values == slave_df[id_col].values):
        raise ValueError("Master and slave IDs are not aligned.")
    if not np.all(master_df[id_col].values == chem_df[id_col].values):
```

```
        raise ValueError("Spectral and chemical IDs are not aligned.")

    return master_df, slave_df, chem_df


#
==========================================================================
====
# 6. Kennard-Stone splitting
#
==========================================================================
====
# Kennard-Stone is used to choose representative samples spread across
the
# spectral space. This avoids selecting all calibration or validation
samples
# from a narrow region of the dataset.

def kennard_stone(X, n_samples):
    """
    Deterministic Kennard-Stone selection based on Euclidean distances in
    standardized spectral space.

    Important reproducibility detail:
    The list of remaining candidate samples is explicitly sorted. This
avoids
    uncontrolled ordering from Python sets and makes tie-breaking stable
when
    samples have equal or almost equal distances.
    """
    X = np.asarray(X, dtype=float)
    if n_samples > X.shape[0]:
        raise ValueError("n_samples cannot exceed the number of available
samples.")

    Xs = StandardScaler().fit_transform(X)
    diff = Xs[:, np.newaxis, :] - Xs[np.newaxis, :, :]
    dist = np.sqrt(np.sum(diff ** 2, axis=2))

    i, j = np.unravel_index(np.argmax(dist), dist.shape)
    selected = [int(i), int(j)]

    # Use sorted indices to make the selection reproducible.
    remaining = sorted(list(set(range(X.shape[0])) - set(selected)))

    while len(selected) < n_samples:
        min_dist = np.min(dist[remaining][:, selected], axis=1)

        # np.argmax returns the first maximum. Because 'remaining' is
sorted,
        # ties are resolved consistently across runs.
        next_position = int(np.argmax(min_dist))
        next_idx = int(remaining[next_position])

        selected.append(next_idx)
        remaining.remove(next_idx)
        remaining = sorted(remaining)

    return np.array(selected, dtype=int), np.array(remaining, dtype=int)
```

```
def sequential_kennard_stone_split(X_split):
    """
    Sequential split performed before preprocessing and modelling.

    The order is:
        20 standardization -> 20 validation -> 11 update -> 50
calibration.
    """
    all_indices = np.arange(X_split.shape[0])

    std_local, rem1_local = kennard_stone(X_split, N_STANDARDIZATION)
    idx_std = all_indices[std_local]
    idx_rem1 = all_indices[rem1_local]

    val_local, rem2_local = kennard_stone(X_split[idx_rem1, :],
N_VALIDATION)
    idx_val = idx_rem1[val_local]
    idx_rem2 = idx_rem1[rem2_local]

    upd_local, rem3_local = kennard_stone(X_split[idx_rem2, :], N_UPDATE)
    idx_upd = idx_rem2[upd_local]
    idx_cal = idx_rem2[rem3_local]

    if len(idx_cal) != N_CALIBRATION:
        raise ValueError(f"Calibration set has {len(idx_cal)} samples,
expected {N_CALIBRATION}.")

    return {
        "standardization": idx_std,
        "calibration": idx_cal,
        "update": idx_upd,
        "validation": idx_val
    }


def build_split_dataframe(sample_ids, split, id_col="ID"):
    """Create a readable table showing the subset assigned to each
sample."""
    split_df = pd.DataFrame({id_col: sample_ids, "Subset":
"not_assigned"})
    split_df.loc[split["standardization"], "Subset"] = "standardization"
    split_df.loc[split["calibration"], "Subset"] = "calibration"
    split_df.loc[split["update"], "Subset"] = "update"
    split_df.loc[split["validation"], "Subset"] = "validation"
    return split_df


def validate_split_counts(split_df):
    """Check that the imported or generated split has the expected subset
sizes."""
    expected_counts = {
        "standardization": N_STANDARDIZATION,
        "calibration": N_CALIBRATION,
        "update": N_UPDATE,
        "validation": N_VALIDATION
    }
    observed_counts = split_df["Subset"].value_counts().to_dict()
```

```
    for subset_name, expected_n in expected_counts.items():
        observed_n = int(observed_counts.get(subset_name, 0))
        if observed_n != expected_n:
            raise ValueError(
                f"The split file has {observed_n} samples in
'{subset_name}', "
                f"but the script expects {expected_n}.\n"
                "Please check the split file or the split-size constants
at the "
                "beginning of the script."
            )


def load_existing_kennard_stone_split(split_path, sample_ids,
id_col="ID"):
    """
    Import the official Kennard-Stone split created by the one-time split
script.

    The file must contain at least two columns:
        - ID: sample identifier;
        - Subset: standardization, calibration, update, or validation.

    The function maps sample IDs from the split file onto the current
aligned
    sample order. This means the main script can use the same split even
if the
    input files are read in a different row order.
    """
    if not os.path.exists(split_path):
        raise FileNotFoundError(
            f"Official Kennard-Stone split file not
found:\n{split_path}\n"
            "Run create_kennard_stone_split_once.py first, or set "
            "USE_EXISTING_KS_SPLIT = False to recalculate the split."
        )

    split_file_df = read_table(split_path)
    split_file_df = clean_column_names(split_file_df)

    required_columns = {id_col, "Subset"}
    missing_columns = required_columns - set(split_file_df.columns)
    if missing_columns:
        raise ValueError(
            f"The split file is missing these required columns:
{sorted(missing_columns)}\n"
            f"Available columns are: {split_file_df.columns.tolist()}"
        )

    split_file_df[id_col] = split_file_df[id_col].astype(str).str.strip()
    split_file_df["Subset"] =
split_file_df["Subset"].astype(str).str.strip().str.lower()

    allowed_subsets = {"standardization", "calibration", "update",
"validation"}
    unknown_subsets = set(split_file_df["Subset"]) - allowed_subsets
    if unknown_subsets:
        raise ValueError(
            f"The split file contains unknown subset names:
{sorted(unknown_subsets)}\n"
```

```
            f"Allowed subset names are: {sorted(allowed_subsets)}"
        )

    if split_file_df[id_col].duplicated().any():
        duplicated_ids =
split_file_df.loc[split_file_df[id_col].duplicated(), id_col].tolist()
        raise ValueError(
            "The split file contains duplicated sample IDs. Examples: "
            f"{duplicated_ids[:10]}"
        )

    current_ids = pd.Series(sample_ids).astype(str).str.strip().tolist()
    current_id_set = set(current_ids)
    split_id_set = set(split_file_df[id_col])

    missing_in_split = sorted(current_id_set - split_id_set)
    extra_in_split = sorted(split_id_set - current_id_set)
    if missing_in_split or extra_in_split:
        raise ValueError(
            "The imported Kennard-Stone split does not match the
currently "
            "aligned samples.\n"
            f"Samples missing in split file: {missing_in_split[:20]}\n"
            f"Samples present only in split file:
{extra_in_split[:20]}\n"
            "Use the same master/slave/chemical files used to create the
split, "
            "or regenerate the official split once."
        )

    subset_by_id = dict(zip(split_file_df[id_col],
split_file_df["Subset"]))
    split_df = pd.DataFrame({id_col: current_ids})
    split_df["Subset"] = split_df[id_col].map(subset_by_id)

    validate_split_counts(split_df)

    split = {
        "standardization": split_df.index[split_df["Subset"] ==
"standardization"].to_numpy(dtype=int),
        "calibration": split_df.index[split_df["Subset"] ==
"calibration"].to_numpy(dtype=int),
        "update": split_df.index[split_df["Subset"] ==
"update"].to_numpy(dtype=int),
        "validation": split_df.index[split_df["Subset"] ==
"validation"].to_numpy(dtype=int)
    }

    return split, split_df


#
==========================================================================
====
# 7. Spectral preprocessing
#
==========================================================================
====
# The SpectralPreprocessor class applies one or more preprocessing steps
to
```

```
# spectra. Examples are SNV, MSC, detrending, smoothing, and derivatives.
# A 'class' is simply a reusable object that remembers fitted parameters,
# such as the MSC reference spectrum.

class SpectralPreprocessor:
    """Spectral preprocessing pipeline."""

    def __init__(self, sequence, params):
        self.sequence = sequence
        self.params = params
        self.msc_reference_ = None

    def fit(self, X):
        """Fit preprocessing parameters, mainly the MSC reference
spectrum."""
        X_fit = np.asarray(X, dtype=float).copy()
        for step, par in zip(self.sequence, self.params):
            if step == "msc":
                self.msc_reference_ = np.mean(X_fit, axis=0)
            X_fit = self._apply_step(X_fit, step, par)
        return self

    def transform(self, X):
        """Apply the fitted preprocessing pipeline."""
        X_trans = np.asarray(X, dtype=float).copy()
        for step, par in zip(self.sequence, self.params):
            X_trans = self._apply_step(X_trans, step, par)
        return X_trans

    def fit_transform(self, X):
        self.fit(X)
        return self.transform(X)

    def _apply_step(self, X, step, par):
        """Apply one preprocessing step."""
        if step == "none":
            return X
        if step == "snv":
            return self._snv(X)
        if step == "msc":
            return self._msc(X)
        if step == "detrend":
            return self._detrend(X)
        if step == "smooth":
            return self._smooth(X, par)
        if step == "sg_derivative":
            return self._sg_derivative(X, par)
        raise ValueError(f"Unknown preprocessing step: {step}")

    @staticmethod
    def _snv(X):
        """Standard Normal Variate correction."""
        mean = np.mean(X, axis=1, keepdims=True)
        std = np.std(X, axis=1, ddof=1, keepdims=True)
        std[std == 0] = 1.0
        return (X - mean) / std

    def _msc(self, X):
        """Multiplicative Scatter Correction using a fitted reference
spectrum."""
```

```
        if self.msc_reference_ is None:
            raise ValueError("MSC reference spectrum has not been
fitted.")
        ref = self.msc_reference_
        X_corr = np.zeros_like(X)
        for i in range(X.shape[0]):
            lr = LinearRegression()
            lr.fit(ref.reshape(-1, 1), X[i, :])
            intercept = lr.intercept_
            slope = lr.coef_[0]
            if slope == 0:
                slope = 1.0
            X_corr[i, :] = (X[i, :] - intercept) / slope
        return X_corr

    @staticmethod
    def _detrend(X):
        """Linear detrending applied to each spectrum."""
        return signal.detrend(X, axis=1, type="linear")

    @staticmethod
    def _smooth(X, par):
        """Savitzky-Golay smoothing."""
        window_length = int(par["window_length"])
        polyorder = int(par["polyorder"])
        if window_length % 2 == 0:
            raise ValueError("Savitzky-Golay window_length must be odd.")
        if window_length <= polyorder:
            raise ValueError("window_length must be greater than
polyorder.")
        return savgol_filter(X, window_length=window_length,
polyorder=polyorder,
                             deriv=0, axis=1, mode="interp")

    @staticmethod
    def _sg_derivative(X, par):
        """Savitzky-Golay derivative; gap keeps one column every 'gap'
columns."""
        window_length = int(par["window_length"])
        polyorder = int(par["polyorder"])
        deriv = int(par["deriv"])
        gap = int(par.get("gap", 1))
        if window_length % 2 == 0:
            raise ValueError("Savitzky-Golay window_length must be odd.")
        if window_length <= polyorder:
            raise ValueError("window_length must be greater than
polyorder.")
        if deriv < 1:
            raise ValueError("Derivative order must be >= 1.")
        X_der = savgol_filter(X, window_length=window_length,
polyorder=polyorder,
                              deriv=deriv, axis=1, mode="interp")
        if gap > 1:
            X_der = X_der[:, ::gap]
        return X_der


#
==========================================================================
====
```

```
# 8. Metrics and PLS modelling
#
=========================================================================
====
# This section contains functions for model evaluation and PLS
regression.
# Metrics such as RMSEP and R2_pred are calculated here.
# PLS components are selected by internal cross-validation, not by the
final
# validation set.

def rmse(y_true, y_pred):
    """Root mean squared error."""
    y_true = np.ravel(y_true)
    y_pred = np.ravel(y_pred)
    return np.sqrt(np.mean((y_true - y_pred) ** 2))


def sep(y_true, y_pred):
    """Standard error of prediction after removing bias."""
    y_true = np.ravel(y_true)
    y_pred = np.ravel(y_pred)
    errors = y_pred - y_true
    bias = np.mean(errors)
    if len(errors) <= 1:
        return np.nan
    return np.sqrt(np.sum((errors - bias) ** 2) / (len(errors) - 1))


def regression_slope_intercept(y_true, y_pred):
    """Slope and intercept of predicted vs observed regression."""
    y_true = np.ravel(y_true).reshape(-1, 1)
    y_pred = np.ravel(y_pred)
    lr = LinearRegression()
    lr.fit(y_true, y_pred)
    return float(lr.coef_[0]), float(lr.intercept_)


def calculate_metrics(y_true, y_pred):
    """Calculate hold-out validation metrics."""
    y_true = np.ravel(y_true)
    y_pred = np.ravel(y_pred)
    rmsep_value = rmse(y_true, y_pred)
    bias_value = np.mean(y_pred - y_true)
    sep_value = sep(y_true, y_pred)
    r2_value = r2_score(y_true, y_pred)
    mae_value = mean_absolute_error(y_true, y_pred)
    rpd_value = np.nan if rmsep_value == 0 else np.std(y_true, ddof=1) /
rmsep_value
    slope, intercept = regression_slope_intercept(y_true, y_pred)
    return {
        "RMSEP": rmsep_value,
        "Bias": bias_value,
        "SEP": sep_value,
        "R2_pred": r2_value,
        "MAE": mae_value,
        "RPD": rpd_value,
        "Slope": slope,
        "Intercept": intercept
    }
```

```
def _cv_predict_pls_with_pls_preprocessing(X_cal_raw, y_cal,
max_components, pls_preproc_cfg):
    """
    Select PLS components using internal CV while fitting PLS
preprocessing
    inside each CV fold.

    This avoids leakage for fit-dependent preprocessing such as MSC.
    """
    X_cal_raw = np.asarray(X_cal_raw, dtype=float)
    y_cal = np.ravel(y_cal)

    max_possible = min(max_components, X_cal_raw.shape[0] - 1,
X_cal_raw.shape[1])
    if max_possible < 1:
        raise ValueError("Not enough samples or variables for PLS.")

    cv = KFold(n_splits=N_SPLITS_CV, shuffle=True,
random_state=RANDOM_STATE)
    rows = []

    for n_comp in range(1, max_possible + 1):
        y_cv = np.zeros_like(y_cal, dtype=float)

        for train_idx, test_idx in cv.split(X_cal_raw):
            X_train_raw = X_cal_raw[train_idx, :]
            X_test_raw = X_cal_raw[test_idx, :]
            y_train = y_cal[train_idx]

            pp = SpectralPreprocessor(pls_preproc_cfg["sequence"],
pls_preproc_cfg["params"])
            pp.fit(X_train_raw)
            X_train = pp.transform(X_train_raw)
            X_test = pp.transform(X_test_raw)

            n_comp_fold = min(n_comp, X_train.shape[0] - 1,
X_train.shape[1])
            pls = PLSRegression(n_components=n_comp_fold, scale=True)
            pls.fit(X_train, y_train)
            y_cv[test_idx] = pls.predict(X_test).ravel()

        rows.append({"PLS_components": n_comp, "RMSECV": rmse(y_cal,
y_cv)})

    cv_df = pd.DataFrame(rows)
    best_row = cv_df.loc[cv_df["RMSECV"].idxmin()]
    return int(best_row["PLS_components"]), float(best_row["RMSECV"]),
cv_df


def fit_final_pls_after_cv(X_cal_raw, y_cal, X_val_raw,
max_pls_components, pls_preproc_cfg):
    """
    Fit final PLS model with a separate PLS-preprocessing stage.

    Steps:
        1. Select the number of PLS components using internal CV.
```

```
        2. Fit the PLS preprocessing on all calibration/update spectra
only.
        3. Transform calibration/update and hold-out validation spectra.
        4. Fit the final PLS model.
        5. Predict the hold-out validation set.
    """
    best_n, best_rmsecv, cv_table =
_cv_predict_pls_with_pls_preprocessing(
        X_cal_raw, y_cal, max_components=max_pls_components,
pls_preproc_cfg=pls_preproc_cfg
    )

    pp_final = SpectralPreprocessor(pls_preproc_cfg["sequence"],
pls_preproc_cfg["params"])
    pp_final.fit(X_cal_raw)
    X_cal = pp_final.transform(X_cal_raw)
    X_val = pp_final.transform(X_val_raw)

    best_n = min(best_n, X_cal.shape[0] - 1, X_cal.shape[1])
    pls = PLSRegression(n_components=best_n, scale=True)
    pls.fit(X_cal, np.ravel(y_cal))

    y_cal_pred = pls.predict(X_cal).ravel()
    y_val_pred = pls.predict(X_val).ravel()

    return {
        "model": pls,
        "pls_preprocessor": pp_final,
        "PLS_components": best_n,
        "RMSECV": best_rmsecv,
        "RMSEC": rmse(y_cal, y_cal_pred),
        "y_val_pred": y_val_pred,
        "cv_table": cv_table,
        "N_variables_after_PLS_preprocessing": X_cal.shape[1]
    }


#
==========================================================================
====
# 9. Calibration transfer methods
#
==========================================================================
====
# These functions implement the different ways to make master spectra
more
# comparable to slave spectra. Bias correction, DS, PDS and Shenk-
Westerhaus
# produce slave-like master spectra. DOP projects spectra into a common
space.

def transfer_bias_correction(X_master_to_transform, X_master_std,
X_slave_std):
    """Wavelength-wise spectral bias correction from master to slave."""
    bias_vector = np.mean(X_slave_std - X_master_std, axis=0)
    return X_master_to_transform + bias_vector


def fit_ds(X_master_std, X_slave_std, alpha=RIDGE_ALPHA):
    """Fit Direct Standardization using ridge regression."""
```

```
    model = Ridge(alpha=alpha, fit_intercept=True)
    model.fit(X_master_std, X_slave_std)
    return model


def apply_ds(model, X_master_to_transform):
    """Apply Direct Standardization."""
    return model.predict(X_master_to_transform)


def fit_pds(X_master_std, X_slave_std, window_size, alpha=RIDGE_ALPHA):
    """Fit Piecewise Direct Standardization."""
    if window_size % 2 == 0:
        raise ValueError("PDS window size must be odd.")
    n_wav = X_master_std.shape[1]
    half = window_size // 2
    models = []
    windows = []
    for j in range(n_wav):
        start = max(0, j - half)
        end = min(n_wav, j + half + 1)
        model = Ridge(alpha=alpha, fit_intercept=True)
        model.fit(X_master_std[:, start:end], X_slave_std[:, j])
        models.append(model)
        windows.append((start, end))
    return models, windows


def apply_pds(pds_model, X_master_to_transform):
    """Apply Piecewise Direct Standardization."""
    models, windows = pds_model
    X_trans = np.zeros((X_master_to_transform.shape[0], len(models)))
    for j, model in enumerate(models):
        start, end = windows[j]
        X_trans[:, j] = model.predict(X_master_to_transform[:,
start:end])
    return X_trans


def fit_shenk_westerhaus(X_master_std, X_slave_std):
    """Fit wavelength-wise Shenk-Westerhaus-type slope/intercept
correction."""
    n_wav = X_master_std.shape[1]
    slopes = np.zeros(n_wav)
    intercepts = np.zeros(n_wav)
    for j in range(n_wav):
        lr = LinearRegression()
        lr.fit(X_master_std[:, j].reshape(-1, 1), X_slave_std[:, j])
        slopes[j] = lr.coef_[0]
        intercepts[j] = lr.intercept_
    return slopes, intercepts


def apply_shenk_westerhaus(sw_model, X_master_to_transform):
    """Apply wavelength-wise Shenk-Westerhaus-type correction."""
    slopes, intercepts = sw_model
    return X_master_to_transform * slopes + intercepts


def fit_dop(X_master_std, X_slave_std, n_dop_components):
```

```
    """Fit DOP projection using paired standardization spectra."""
    D = X_slave_std - X_master_std
    pca = PCA(n_components=n_dop_components)
    pca.fit(D)
    P = pca.components_.T
    projection_matrix = np.eye(P.shape[0]) - P @ P.T
    return projection_matrix, pca


def apply_dop(dop_model, X):
    """Apply DOP projection."""
    projection_matrix, _ = dop_model
    return X @ projection_matrix


def fit_transfer_preprocessor(X_master_raw, X_slave_raw, idx_std,
idx_cal, idx_upd, transfer_cfg):
    """
    Fit the transfer preprocessing pipeline without using hold-out
validation samples.

    This preprocessing is used only for estimating/applying transfer
functions.
    """
    fit_indices = np.concatenate([idx_std, idx_cal, idx_upd])
    X_fit = np.vstack([X_master_raw[fit_indices, :],
X_slave_raw[fit_indices, :]])
    pp = SpectralPreprocessor(transfer_cfg["sequence"],
transfer_cfg["params"])
    pp.fit(X_fit)
    return pp


#
==========================================================================
====
# 10. Method evaluation functions
#
==========================================================================
====
# These functions combine transfer, preprocessing, PLS modelling and
validation.
# Each function returns both predictions and numerical performance
metrics.

def evaluate_master_benchmark(Xm_cal_raw, Xm_upd_raw, Xm_val_raw, y_cal,
y_upd, y_val,
                              max_pls_components, pls_cfg):
    """Evaluate original master calibration benchmark using PLS
preprocessing only."""
    X_model_cal_raw = np.vstack([Xm_cal_raw, Xm_upd_raw])
    y_model_cal = np.concatenate([y_cal, y_upd])

    pls_result = fit_final_pls_after_cv(
        X_model_cal_raw, y_model_cal, Xm_val_raw, max_pls_components,
pls_cfg
    )
    metrics = calculate_metrics(y_val, pls_result["y_val_pred"])

    return {
```

```
        "Method": "Original master benchmark",
        "Method_detail": "PLS preprocessing only; no transfer
preprocessing",
        "DOP_components": np.nan,
        "Calibration_matrix_description": "50 original master + 11
original master",
        "Calibration_samples": X_model_cal_raw.shape[0],
        "PLS_components": pls_result["PLS_components"],
        "N_variables_after_PLS_preprocessing":
pls_result["N_variables_after_PLS_preprocessing"],
        "RMSEC": pls_result["RMSEC"],
        "RMSECV": pls_result["RMSECV"],
        "y_val_pred": pls_result["y_val_pred"],
        **metrics
    }


def evaluate_standard_transfer_method(method_name, Xm_std_t, Xs_std_t,
Xm_cal_t, Xs_upd_t, Xs_val_t,
                                      y_cal, y_upd, y_val,
max_pls_components, pls_cfg,
                                      pds_window=None):
    """
    Evaluate bias correction, DS, PDS or Shenk-Westerhaus.

    Inputs are already transfer-preprocessed. After the transfer step, a
separate
    PLS preprocessing is fitted on the 61-sample calibration/update
dataset.
    """
    if method_name == "Spectral bias correction":
        Xm_cal_trans = transfer_bias_correction(Xm_cal_t, Xm_std_t,
Xs_std_t)
        method_detail = ""
    elif method_name == "DS":
        ds_model = fit_ds(Xm_std_t, Xs_std_t)
        Xm_cal_trans = apply_ds(ds_model, Xm_cal_t)
        method_detail = ""
    elif method_name == "PDS":
        pds_model = fit_pds(Xm_std_t, Xs_std_t, window_size=pds_window)
        Xm_cal_trans = apply_pds(pds_model, Xm_cal_t)
        method_detail = f"window={pds_window}"
    elif method_name == "Shenk-Westerhaus":
        sw_model = fit_shenk_westerhaus(Xm_std_t, Xs_std_t)
        Xm_cal_trans = apply_shenk_westerhaus(sw_model, Xm_cal_t)
        method_detail = ""
    else:
        raise ValueError(f"Unknown transfer method: {method_name}")

    X_model_cal_raw = np.vstack([Xm_cal_trans, Xs_upd_t])
    y_model_cal = np.concatenate([y_cal, y_upd])

    pls_result = fit_final_pls_after_cv(
        X_model_cal_raw, y_model_cal, Xs_val_t, max_pls_components,
pls_cfg
    )
    metrics = calculate_metrics(y_val, pls_result["y_val_pred"])

    return {
        "Method": method_name,
```

```
        "Method_detail": method_detail,
        "DOP_components": np.nan,
        "Calibration_matrix_description": "50 transformed master + 11
original slave, followed by PLS preprocessing",
        "Calibration_samples": X_model_cal_raw.shape[0],
        "PLS_components": pls_result["PLS_components"],
        "N_variables_after_PLS_preprocessing":
pls_result["N_variables_after_PLS_preprocessing"],
        "RMSEC": pls_result["RMSEC"],
        "RMSECV": pls_result["RMSECV"],
        "y_val_pred": pls_result["y_val_pred"],
        **metrics
    }


def evaluate_dop_method(Xm_std_t, Xs_std_t, Xm_cal_t, Xs_upd_t, Xs_val_t,
                        y_cal, y_upd, y_val, max_pls_components,
max_dop_components, pls_cfg):
    """
    Evaluate DOP with DOP components selected by internal PLS CV.

    DOP uses transfer-preprocessed spectra. For each candidate DOP
component
    number, the projected calibration/update set is passed to the PLS
stage,
    where PLS preprocessing and PLS component selection are performed by
    internal CV only.
    """
    y_model_cal = np.concatenate([y_cal, y_upd])
    candidate_rows = []
    candidate_objects = []

    for n_dop in range(1, max_dop_components + 1):
        if n_dop >= Xm_std_t.shape[0] or n_dop >= Xm_std_t.shape[1]:
            continue

        dop_model = fit_dop(Xm_std_t, Xs_std_t, n_dop_components=n_dop)
        Xm_cal_dop = apply_dop(dop_model, Xm_cal_t)
        Xs_upd_dop = apply_dop(dop_model, Xs_upd_t)
        X_model_cal_raw = np.vstack([Xm_cal_dop, Xs_upd_dop])

        best_pls, rmsecv, cv_table =
_cv_predict_pls_with_pls_preprocessing(
            X_model_cal_raw, y_model_cal,
max_components=max_pls_components, pls_preproc_cfg=pls_cfg
        )

        candidate_rows.append({
            "DOP_components": n_dop,
            "Best_PLS_components": best_pls,
            "Internal_RMSECV": rmsecv
        })
        candidate_objects.append({
            "DOP_components": n_dop,
            "DOP_model": dop_model,
            "X_model_cal_raw": X_model_cal_raw,
            "Best_PLS_components": best_pls,
            "RMSECV": rmsecv,
            "CV_table": cv_table
        })
```

```
    dop_cv_df = pd.DataFrame(candidate_rows)
    if dop_cv_df.empty:
        raise ValueError("No valid DOP candidate was generated.")

    best_idx = dop_cv_df["Internal_RMSECV"].idxmin()
    best_candidate = candidate_objects[best_idx]

    dop_model = best_candidate["DOP_model"]
    X_model_cal_raw = best_candidate["X_model_cal_raw"]
    Xs_val_dop = apply_dop(dop_model, Xs_val_t)

    pls_result = fit_final_pls_after_cv(
        X_model_cal_raw, y_model_cal, Xs_val_dop, max_pls_components,
pls_cfg
    )
    metrics = calculate_metrics(y_val, pls_result["y_val_pred"])

    return {
        "Method": "DOP",
        "Method_detail": "DOP components selected by internal PLS CV;
separate PLS preprocessing applied after projection",
        "DOP_components": int(best_candidate["DOP_components"]),
        "Calibration_matrix_description": "50 projected master + 11
projected slave, followed by PLS preprocessing",
        "Calibration_samples": X_model_cal_raw.shape[0],
        "PLS_components": pls_result["PLS_components"],
        "N_variables_after_PLS_preprocessing":
pls_result["N_variables_after_PLS_preprocessing"],
        "RMSEC": pls_result["RMSEC"],
        "RMSECV": float(best_candidate["RMSECV"]),
        "y_val_pred": pls_result["y_val_pred"],
        "DOP_cv_table": dop_cv_df,
        **metrics
    }


#
==========================================================================
====
# 11. Author-parameter tables for Excel export
#
==========================================================================
====
# These functions convert the user's choices into tables.
# They make the Excel output traceable: later, you can see exactly which
# preprocessing sequences and parameter grids were tested.

def configs_to_dataframe(configs, stage_name):
    """Convert preprocessing configurations to a table."""
    rows = []
    for i, cfg in enumerate(configs, start=1):
        rows.append({
            "Stage": stage_name,
            "Configuration_ID": i,
            "Preprocessing": cfg["label"],
            "Sequence": " + ".join(cfg["sequence"]),
            "Parameters_JSON": json.dumps(cfg["params"])
        })
    return pd.DataFrame(rows)
```

```
def grid_to_dataframe(grid, stage_name):
    """Convert preprocessing parameter grid to a table."""
    rows = []
    for step, params_list in grid.items():
        for i, params in enumerate(params_list, start=1):
            rows.append({
                "Stage": stage_name,
                "Preprocessing_step": step,
                "Grid_row": i,
                "Parameters_JSON": json.dumps(params)
            })
    return pd.DataFrame(rows)


#
==========================================================================
====
# 12. Main workflow
#
==========================================================================
====
# The main() function runs the complete analysis in the correct order.
# It is intentionally written as a sequence of clearly separated steps so
that
# non-expert users can follow the workflow from input files to final
outputs.

def main():
    """Run the full interactive calibration transfer workflow."""
    print("\n" + "=" * 90)
    print("Interactive calibration transfer analysis: master -> slave")
    print("Separate preprocessing for transfer and PLS stages")
    print("=" * 90)

    # Step 1: decide which folder will be used for reading and saving
files.
    # If DATA_DIR is wrong, the script falls back to the script folder.
    data_dir = resolve_data_dir(DATA_DIR)

    # Step 2: ask the user what should be tested.
    # The answers define the experimental modelling grid.
    # Nothing is modelled yet at this stage.
    # Ask the user for transfer preprocessing, PLS preprocessing and
model grids.
    transfer_sequences = ask_preprocessing_sequences(
        stage_name="TRANSFER",
        default_sequences="none, snv, msc, snv+smooth,
snv+sg_derivative+smooth, msc+smooth, msc+sg_derivative+smooth"
    )
    transfer_parameter_grid =
ask_preprocessing_parameter_grid(transfer_sequences,
stage_name="TRANSFER")
    transfer_configs = expand_preprocessing_grid(transfer_sequences,
transfer_parameter_grid)

    pls_sequences = ask_preprocessing_sequences(
        stage_name="PLS",
```

```
        default_sequences="none, snv, msc, snv+smooth,
snv+sg_derivative+smooth, msc+smooth, msc+sg_derivative+smooth"
    )
    pls_parameter_grid = ask_preprocessing_parameter_grid(pls_sequences,
stage_name="PLS")
    pls_configs = expand_preprocessing_grid(pls_sequences,
pls_parameter_grid)

    model_grid = ask_model_parameter_grid()
    max_pls_components = model_grid["max_pls_components"]
    max_dop_components = model_grid["max_dop_components"]
    pds_windows = model_grid["pds_windows"]

    print("\nTransfer preprocessing configurations generated:")
    for i, cfg in enumerate(transfer_configs, start=1):
        print(f"TRANSFER {i:03d}: {cfg['label']}")

    print("\nPLS preprocessing configurations generated:")
    for i, cfg in enumerate(pls_configs, start=1):
        print(f"PLS {i:03d}: {cfg['label']}")

    print("\nModel grid:")
    print(f"Maximum PLS components: {max_pls_components}")
    print(f"Maximum DOP components: {max_dop_components}")
    print(f"PDS windows: {pds_windows}")

    # Step 3: load the three input files into pandas tables.
    # At this point, no calculations are performed; the data are simply
read.
    # Load spectral and chemical data.
    # The exact file paths and the first columns are printed to simplify
    # debugging when a wrong folder, delimiter, or ID column name is
used.
    master_path = os.path.join(data_dir, MASTER_FILE)
    slave_path = os.path.join(data_dir, SLAVE_FILE)
    chemical_path = os.path.join(data_dir, CHEMICAL_FILE)

    print("\nFiles actually read:")
    print(f"Master:   {master_path}")
    print(f"Slave:    {slave_path}")
    print(f"Chemical: {chemical_path}")

    master_df = read_table(master_path)
    slave_df = read_table(slave_path)
    chemical_df = read_table(chemical_path)

    print("\nFirst columns detected after import:")
    print(f"Master columns:   {master_df.columns.tolist()[:30]}")
    print(f"Slave columns:    {slave_df.columns.tolist()[:30]}")
    print(f"Chemical columns: {chemical_df.columns.tolist()[:30]}")

    # Step 4: align samples by ID.
    # This prevents comparing the spectrum of one sample with the
chemistry of another.
    master_df, slave_df, chemical_df = align_master_slave_chemical(
        master_df, slave_df, chemical_df, id_col=ID_COL
    )

    # Step 5: identify the wavelength columns.
    # Only columns with numeric names are treated as wavelengths.
```

```
    master_cols = get_spectral_columns(master_df, id_col=ID_COL)
    slave_cols = get_spectral_columns(slave_df, id_col=ID_COL)
    if master_cols != slave_cols:
        raise ValueError("Master and slave spectral columns are not
identical.")

    wavelengths = np.array([float(c) for c in master_cols])
    X_master_raw = master_df[master_cols].values.astype(float)
    X_slave_raw = slave_df[slave_cols].values.astype(float)
    sample_ids = master_df[ID_COL].values.astype(str)

    expected_total = N_STANDARDIZATION + N_CALIBRATION + N_UPDATE +
N_VALIDATION
    actual_total = X_master_raw.shape[0]
    if actual_total != expected_total:
        raise ValueError(
            f"The aligned dataset contains {actual_total} samples, but
the requested split sizes sum to {expected_total}.\n"
            f"Current split sizes are:
standardization={N_STANDARDIZATION}, calibration={N_CALIBRATION}, "
            f"update={N_UPDATE}, validation={N_VALIDATION}.\n"
            "Please update the split sizes at the beginning of the script
or use input files with the expected number of samples."
        )

    print("\nData loaded successfully.")
    print(f"Number of samples: {X_master_raw.shape[0]}")
    print(f"Number of wavelengths: {X_master_raw.shape[1]}")
    print(f"Spectral range: {wavelengths.min():.0f}-
{wavelengths.max():.0f} nm")

    # Step 6: import the official Kennard-Stone split.
    # For reproducibility, the split should be created once with the
separate
    # script create_kennard_stone_split_once.py and then reused here.
    # This avoids any accidental change of
standardization/calibration/update/
    # validation samples across repeated runs of the modelling script.
    X_split = np.hstack([X_master_raw, X_slave_raw])

    if USE_EXISTING_KS_SPLIT:
        split_path = os.path.join(data_dir, KS_SPLIT_FILE)
        split, split_df = load_existing_kennard_stone_split(
            split_path, sample_ids, id_col=ID_COL
        )
        print("\nOfficial Kennard-Stone split imported successfully.")
        print(f"Split file used: {split_path}")
    else:
        split = sequential_kennard_stone_split(X_split)
        split_df = build_split_dataframe(sample_ids, split,
id_col=ID_COL)
        split_df.to_csv(os.path.join(data_dir, OUTPUT_SPLIT),
index=False)
        print("\nKennard-Stone split recalculated and saved.")

    idx_std = split["standardization"]
    idx_cal = split["calibration"]
    idx_upd = split["update"]
    idx_val = split["validation"]
```

```
    print(split_df["Subset"].value_counts())

    # Step 7: create empty containers where results will be accumulated.
    # Lists are used because results are appended progressively during
loops.
    all_results = []
    all_predictions = []
    all_dop_cv = []

    # -------------------------------------------------------------------
------
    # Step 8: run the master benchmark.
    # This is the reference model built and validated using only the
master instrument.
    # It does not use any master -> slave transfer method.
    # Master benchmark: depends on PLS preprocessing only, not transfer
preprocessing.
    # -------------------------------------------------------------------
------
    for pls_id, pls_cfg in enumerate(pls_configs, start=1):
        pls_label = pls_cfg["label"]
        print("\n" + "=" * 100)
        print(f"Master benchmark - PLS preprocessing
{pls_id}/{len(pls_configs)}: {pls_label}")
        print("=" * 100)

        Xm_cal_raw = X_master_raw[idx_cal, :]
        Xm_upd_raw = X_master_raw[idx_upd, :]
        Xm_val_raw = X_master_raw[idx_val, :]

        for response_col in RESPONSE_COLUMNS:
            if response_col not in chemical_df.columns:
                print(f"WARNING: response column not found and skipped:
{response_col}")
                continue

            y_all = chemical_df[response_col].values.astype(float)
            y_cal = y_all[idx_cal]
            y_upd = y_all[idx_upd]
            y_val = y_all[idx_val]

            master_res = evaluate_master_benchmark(
                Xm_cal_raw, Xm_upd_raw, Xm_val_raw, y_cal, y_upd, y_val,
                max_pls_components, pls_cfg
            )

            all_results.append({
                "Response": response_col,
                "Transfer_preprocessing": "not_applicable",
                "Transfer_preprocessing_sequence": "not_applicable",
                "Transfer_preprocessing_parameters": "not_applicable",
                "PLS_preprocessing": pls_label,
                "PLS_preprocessing_sequence": " +
".join(pls_cfg["sequence"]),
                "PLS_preprocessing_parameters":
json.dumps(pls_cfg["params"]),
                "N_variables_after_transfer_preprocessing":
X_master_raw.shape[1],
                "Transfer_direction": "None",
```

```
                **{k: v for k, v in master_res.items() if k not in
["y_val_pred"]}
            })

            all_predictions.append(pd.DataFrame({
                ID_COL: sample_ids[idx_val],
                "Response": response_col,
                "Transfer_preprocessing": "not_applicable",
                "PLS_preprocessing": pls_label,
                "Method": "Original master benchmark",
                "Observed": y_val,
                "Predicted": master_res["y_val_pred"]
            }))

    # ------------------------------------------------------------------
------
    # Step 9: run all slave-compatible transfer models.
    # For each transfer preprocessing, each PLS preprocessing, each
response variable,
    # and each transfer method, the script builds and validates a model.
    # Transfer models: depend on both transfer preprocessing and PLS
preprocessing.
    # ------------------------------------------------------------------
------
    for transfer_id, transfer_cfg in enumerate(transfer_configs,
start=1):
        transfer_label = transfer_cfg["label"]
        print("\n" + "=" * 100)
        print(f"Transfer preprocessing
{transfer_id}/{len(transfer_configs)}: {transfer_label}")
        print("=" * 100)

        # Fit transfer preprocessing without using external validation
samples.
        transfer_pp = fit_transfer_preprocessor(
            X_master_raw, X_slave_raw, idx_std, idx_cal, idx_upd,
transfer_cfg
        )
        X_master_t = transfer_pp.transform(X_master_raw)
        X_slave_t = transfer_pp.transform(X_slave_raw)
        n_variables_after_transfer = X_master_t.shape[1]

        Xm_std_t = X_master_t[idx_std, :]
        Xs_std_t = X_slave_t[idx_std, :]
        Xm_cal_t = X_master_t[idx_cal, :]
        Xs_upd_t = X_slave_t[idx_upd, :]
        Xs_val_t = X_slave_t[idx_val, :]

        for pls_id, pls_cfg in enumerate(pls_configs, start=1):
            pls_label = pls_cfg["label"]
            print(f"  PLS preprocessing {pls_id}/{len(pls_configs)}:
{pls_label}")

            for response_col in RESPONSE_COLUMNS:
                if response_col not in chemical_df.columns:
                    print(f"WARNING: response column not found and
skipped: {response_col}")
                    continue

                print(f"    Analysing response: {response_col}")
```

```
                y_all = chemical_df[response_col].values.astype(float)
                y_cal = y_all[idx_cal]
                y_upd = y_all[idx_upd]
                y_val = y_all[idx_val]

                # Standard transfer methods.
                for method_name in ["Spectral bias correction", "DS",
"Shenk-Westerhaus"]:
                    res = evaluate_standard_transfer_method(
                        method_name, Xm_std_t, Xs_std_t, Xm_cal_t,
Xs_upd_t, Xs_val_t,
                        y_cal, y_upd, y_val, max_pls_components, pls_cfg
                    )
                    all_results.append({
                        "Response": response_col,
                        "Transfer_preprocessing": transfer_label,
                        "Transfer_preprocessing_sequence": " +
".join(transfer_cfg["sequence"]),
                        "Transfer_preprocessing_parameters":
json.dumps(transfer_cfg["params"]),
                        "PLS_preprocessing": pls_label,
                        "PLS_preprocessing_sequence": " +
".join(pls_cfg["sequence"]),
                        "PLS_preprocessing_parameters":
json.dumps(pls_cfg["params"]),
                        "N_variables_after_transfer_preprocessing":
n_variables_after_transfer,
                        "Transfer_direction": "Master -> Slave",
                        **{k: v for k, v in res.items() if k not in
["y_val_pred"]}
                    })
                    all_predictions.append(pd.DataFrame({
                        ID_COL: sample_ids[idx_val],
                        "Response": response_col,
                        "Transfer_preprocessing": transfer_label,
                        "PLS_preprocessing": pls_label,
                        "Method": method_name,
                        "Observed": y_val,
                        "Predicted": res["y_val_pred"]
                    }))

                # PDS with user-defined windows.
                for pds_window in pds_windows:
                    res = evaluate_standard_transfer_method(
                        "PDS", Xm_std_t, Xs_std_t, Xm_cal_t, Xs_upd_t,
Xs_val_t,
                        y_cal, y_upd, y_val, max_pls_components, pls_cfg,
                        pds_window=pds_window
                    )
                    all_results.append({
                        "Response": response_col,
                        "Transfer_preprocessing": transfer_label,
                        "Transfer_preprocessing_sequence": " +
".join(transfer_cfg["sequence"]),
                        "Transfer_preprocessing_parameters":
json.dumps(transfer_cfg["params"]),
                        "PLS_preprocessing": pls_label,
                        "PLS_preprocessing_sequence": " +
".join(pls_cfg["sequence"]),
```

```
                        "PLS_preprocessing_parameters":
json.dumps(pls_cfg["params"]),
                        "N_variables_after_transfer_preprocessing":
n_variables_after_transfer,
                        "Transfer_direction": "Master -> Slave",
                        **{k: v for k, v in res.items() if k not in
["y_val_pred"]}
                    })
                    all_predictions.append(pd.DataFrame({
                        ID_COL: sample_ids[idx_val],
                        "Response": response_col,
                        "Transfer_preprocessing": transfer_label,
                        "PLS_preprocessing": pls_label,
                        "Method": f"PDS window={pds_window}",
                        "Observed": y_val,
                        "Predicted": res["y_val_pred"]
                    }))

                # DOP with internal PLS CV selection of DOP components.
                dop_res = evaluate_dop_method(
                    Xm_std_t, Xs_std_t, Xm_cal_t, Xs_upd_t, Xs_val_t,
                    y_cal, y_upd, y_val,
                    max_pls_components=max_pls_components,
                    max_dop_components=max_dop_components,
                    pls_cfg=pls_cfg
                )
                all_results.append({
                    "Response": response_col,
                    "Transfer_preprocessing": transfer_label,
                    "Transfer_preprocessing_sequence": " +
".join(transfer_cfg["sequence"]),
                    "Transfer_preprocessing_parameters":
json.dumps(transfer_cfg["params"]),
                    "PLS_preprocessing": pls_label,
                    "PLS_preprocessing_sequence": " +
".join(pls_cfg["sequence"]),
                    "PLS_preprocessing_parameters":
json.dumps(pls_cfg["params"]),
                    "N_variables_after_transfer_preprocessing":
n_variables_after_transfer,
                    "Transfer_direction": "Common projected space",
                    **{k: v for k, v in dop_res.items() if k not in
["y_val_pred", "DOP_cv_table"]}
                })
                all_predictions.append(pd.DataFrame({
                    ID_COL: sample_ids[idx_val],
                    "Response": response_col,
                    "Transfer_preprocessing": transfer_label,
                    "PLS_preprocessing": pls_label,
                    "Method": "DOP",
                    "Observed": y_val,
                    "Predicted": dop_res["y_val_pred"]
                }))
                dop_cv_table = dop_res["DOP_cv_table"].copy()
                dop_cv_table["Response"] = response_col
                dop_cv_table["Transfer_preprocessing"] = transfer_label
                dop_cv_table["PLS_preprocessing"] = pls_label
                all_dop_cv.append(dop_cv_table)
```

```
    # ---------------------------------------------------------------------
------
    # Step 10: collect all results and export them.
    # Excel sheets are created to make comparison and traceability
easier.
    # Export results.
    # ---------------------------------------------------------------------
------
    results_df = pd.DataFrame(all_results)
    predictions_df = pd.concat(all_predictions, axis=0,
ignore_index=True)
    dop_cv_df = pd.concat(all_dop_cv, axis=0, ignore_index=True) if
all_dop_cv else pd.DataFrame()

    results_df = results_df.sort_values(by=["Response", "RMSEP"],
ascending=[True, True]).reset_index(drop=True)

    transferred_df = results_df[results_df["Method"] != "Original master
benchmark"].copy()
    best_transfer_df = (
        transferred_df.sort_values(["Response", "RMSEP"],
ascending=[True, True])
        .groupby("Response", as_index=False)
        .first()
    )

    master_benchmark_df = results_df[results_df["Method"] == "Original
master benchmark"].copy()
    best_master_df = (
        master_benchmark_df.sort_values(["Response", "RMSEP"],
ascending=[True, True])
        .groupby("Response", as_index=False)
        .first()
    )

    rmsep_matrix = results_df.pivot_table(
        index=["Response", "Transfer_preprocessing",
"PLS_preprocessing"],
        columns="Method", values="RMSEP", aggfunc="min"
    ).reset_index()
    r2_matrix = results_df.pivot_table(
        index=["Response", "Transfer_preprocessing",
"PLS_preprocessing"],
        columns="Method", values="R2_pred", aggfunc="max"
    ).reset_index()
    pcs_matrix = results_df.pivot_table(
        index=["Response", "Transfer_preprocessing",
"PLS_preprocessing"],
        columns="Method", values="PLS_components", aggfunc="first"
    ).reset_index()

    transfer_cfg_df = configs_to_dataframe(transfer_configs, "transfer")
    pls_cfg_df = configs_to_dataframe(pls_configs, "pls")
    preproc_cfg_df = pd.concat([transfer_cfg_df, pls_cfg_df], axis=0,
ignore_index=True)

    transfer_grid_df = grid_to_dataframe(transfer_parameter_grid,
"transfer")
    pls_grid_df = grid_to_dataframe(pls_parameter_grid, "pls")
```

```
    preproc_grid_df = pd.concat([transfer_grid_df, pls_grid_df], axis=0,
ignore_index=True)

    model_grid_df = pd.DataFrame([
        {"Parameter": "max_pls_components", "Value": max_pls_components},
        {"Parameter": "max_dop_components", "Value": max_dop_components},
        {"Parameter": "pds_windows", "Value": json.dumps(pds_windows)},
        {"Parameter": "ridge_alpha", "Value": RIDGE_ALPHA},
        {"Parameter": "n_splits_cv", "Value": N_SPLITS_CV},
        {"Parameter": "random_state", "Value": RANDOM_STATE}
    ])

    author_parameters_df = pd.DataFrame([
        {"Parameter": "DATA_DIR", "Value": data_dir},
        {"Parameter": "MASTER_FILE", "Value": MASTER_FILE},
        {"Parameter": "SLAVE_FILE", "Value": SLAVE_FILE},
        {"Parameter": "CHEMICAL_FILE", "Value": CHEMICAL_FILE},
        {"Parameter": "N_STANDARDIZATION", "Value": N_STANDARDIZATION},
        {"Parameter": "N_CALIBRATION", "Value": N_CALIBRATION},
        {"Parameter": "N_UPDATE", "Value": N_UPDATE},
        {"Parameter": "N_VALIDATION", "Value": N_VALIDATION},
        {"Parameter": "OUTPUT_EXCEL", "Value": OUTPUT_EXCEL},
        {"Parameter": "OUTPUT_CSV", "Value": OUTPUT_CSV},
        {"Parameter": "OUTPUT_PREDICTIONS_CSV", "Value":
OUTPUT_PREDICTIONS_CSV},
        {"Parameter": "OUTPUT_WORKSPACE", "Value": OUTPUT_WORKSPACE},
        {"Parameter": "KS_SPLIT_FILE", "Value": KS_SPLIT_FILE},
        {"Parameter": "USE_EXISTING_KS_SPLIT", "Value":
USE_EXISTING_KS_SPLIT}
    ])

    response_columns_df = pd.DataFrame({"Response": RESPONSE_COLUMNS})
    transfer_methods_df = pd.DataFrame({
        "Transfer_method": [
            "Original master benchmark",
            "Spectral bias correction",
            "DS",
            "PDS",
            "Shenk-Westerhaus",
            "DOP"
        ]
    })

    output_excel_path = os.path.join(data_dir, OUTPUT_EXCEL)
    output_csv_path = os.path.join(data_dir, OUTPUT_CSV)
    output_predictions_csv_path = os.path.join(data_dir,
OUTPUT_PREDICTIONS_CSV)
    output_workspace_path = os.path.join(data_dir, OUTPUT_WORKSPACE)

    # -----------------------------------------------------------------
--
    # Safety export before Excel creation
    # -----------------------------------------------------------------
--
    # These objects are made global so that they remain visible in
Spyder's
    # Variable Explorer and can be accessed directly from the IPython
console
    # after the script has finished or even if a later export step fails.
    globals().update({
```

```
        "results_df": results_df,
        "predictions_df": predictions_df,
        "dop_cv_df": dop_cv_df,
        "best_transfer_df": best_transfer_df,
        "best_master_df": best_master_df,
        "rmsep_matrix": rmsep_matrix,
        "r2_matrix": r2_matrix,
        "pcs_matrix": pcs_matrix,
        "split_df": split_df,
        "preproc_grid_df": preproc_grid_df,
        "preproc_cfg_df": preproc_cfg_df,
        "model_grid_df": model_grid_df,
        "author_parameters_df": author_parameters_df,
        "response_columns_df": response_columns_df,
        "transfer_methods_df": transfer_methods_df,
        "data_dir": data_dir,
        "output_csv_path": output_csv_path,
        "output_predictions_csv_path": output_predictions_csv_path,
        "output_excel_path": output_excel_path,
        "output_workspace_path": output_workspace_path
    })

    # Save the two most important large tables immediately as CSV files.
    # CSV files do not have Excel's limit of 1,048,576 rows per sheet.
    # Therefore, even if the later Excel export fails, these results are
kept.
    results_df.to_csv(output_csv_path, index=False)
    predictions_df.to_csv(output_predictions_csv_path, index=False)

    # Save a Python workspace file. This is useful for reopening the main
    # result objects later in Python/Spyder without rerunning the full
analysis.
    workspace = {
        "results_df": results_df,
        "predictions_df": predictions_df,
        "dop_cv_df": dop_cv_df,
        "best_transfer_df": best_transfer_df,
        "best_master_df": best_master_df,
        "rmsep_matrix": rmsep_matrix,
        "r2_matrix": r2_matrix,
        "pcs_matrix": pcs_matrix,
        "split_df": split_df,
        "preproc_grid_df": preproc_grid_df,
        "preproc_cfg_df": preproc_cfg_df,
        "model_grid_df": model_grid_df,
        "author_parameters_df": author_parameters_df,
        "response_columns_df": response_columns_df,
        "transfer_methods_df": transfer_methods_df
    }
    with open(output_workspace_path, "wb") as f:
        pickle.dump(workspace, f)

    # Excel is used only for summary tables that fit within Excel limits.
    # The full validation predictions are not written to Excel because
this
    # table can exceed the maximum number of rows allowed in one Excel
sheet.
    predictions_note_df = pd.DataFrame([
        {
```

```
            "Note": "The full validation predictions table was saved as a
separate CSV file because it may exceed Excel row limits.",
            "CSV_file": OUTPUT_PREDICTIONS_CSV,
            "Rows": predictions_df.shape[0],
            "Columns": predictions_df.shape[1]
        }
    ])

    with pd.ExcelWriter(output_excel_path, engine="openpyxl") as writer:
        best_transfer_df.to_excel(writer,
sheet_name="Best_Slave_transfer", index=False)
        best_master_df.to_excel(writer,
sheet_name="Best_Master_benchmark", index=False)
        results_df.to_excel(writer, sheet_name="All_results",
index=False)
        rmsep_matrix.to_excel(writer, sheet_name="RMSEP_matrix",
index=False)
        r2_matrix.to_excel(writer, sheet_name="R2_matrix", index=False)
        pcs_matrix.to_excel(writer, sheet_name="PLS_PC_matrix",
index=False)
        predictions_note_df.to_excel(writer,
sheet_name="Validation_pred_note", index=False)
        split_df.to_excel(writer, sheet_name="Kennard_Stone_split",
index=False)
        if not dop_cv_df.empty:
            dop_cv_df.to_excel(writer, sheet_name="DOP_internal_CV",
index=False)
        author_parameters_df.to_excel(writer,
sheet_name="Author_parameters", index=False)
        preproc_grid_df.to_excel(writer, sheet_name="Preproc_grid",
index=False)
        preproc_cfg_df.to_excel(writer,
sheet_name="Preproc_configurations", index=False)
        model_grid_df.to_excel(writer, sheet_name="Model_grid",
index=False)
        response_columns_df.to_excel(writer,
sheet_name="Response_columns", index=False)
        transfer_methods_df.to_excel(writer,
sheet_name="Transfer_methods", index=False)

    print("\nAnalysis completed.")
    print(f"CSV results saved to:\n{output_csv_path}")
    print(f"Full validation predictions CSV saved
to:\n{output_predictions_csv_path}")
    print(f"Python workspace saved to:\n{output_workspace_path}")
    print(f"Excel results saved to:\n{output_excel_path}")


#
==========================================================================
====
# 13. Run script
#
==========================================================================
====

if __name__ == "__main__":
    main()
```
