# Supplementary material for: From Benchtop to Handheld Systems: Calibration Transfer for On-Site NIR System Butter Quality Monitoring
Source: Sensors (Basel). 2026 Sep 26;26(19):6110. doi: 10.3390/s26196110 (PMC13667566; doi:10.3390/s26196110)
Supplement: Supplementary file 1 [file sensors-26-06110-s001.zip › sensors-4504687-supplementary S2.pdf]

Supplementary File S2: Python code for the reduced top-five ensemble analysis

```
# -*- coding: utf-8 -*-
"""
====================

Reduced top-five ensemble workflow for calibration transfer from the DS2500 (DS25)
master instrument to Aurora, ISC, and JDSU handheld NIR instruments.

PURPOSE
-------
This script reproduces the reduced top-five ensemble analysis described in the
manuscript. It uses the workspace files generated by Supplementary File S1.

For each response variable and target instrument, the script:
1. excludes the original master benchmark;
2. ranks all transferred configurations using five hold-out validation criteria:
    - lower RMSEP;
    - higher R2_pred;
    - lower absolute bias;
    - higher RPD;
    - slope closer to 1;
3. sums the five ranks and retains the five configurations with the lowest
   composite rank;
4. averages the five predictions sample-by-sample across the 19 hold-out
   validation samples;
5. recalculates ensemble RMSEP, R2_pred, and RPD from the averaged predictions;
6. compares the ensemble RMSEP with the lowest RMSEP among the five selected
   configurations;
```

7. exports the response-level results, selected configurations, sample-level
   ensemble predictions, and the summary used for Table 9.

IMPORTANT INTERPRETATION

The same hold-out validation set is used both to rank candidate configurations and to assess the resulting ensemble. Therefore, the resulting ensemble metrics are exploratory/descriptive and are not fully independent estimates of generalisation performance.

INPUT

Three Python workspace files generated by Supplementary File S1:

   Aurora_calibration_transfer_workspace.pkl

   ISC_calibration_transfer_workspace.pkl

   JDSU_calibration_transfer_workspace.pkl

OUTPUT

------

reduced_top_five_ensemble_results_32responses.xlsx

The workbook contains:

   Table9_summary

   Response_metrics

   Selected_top5

   Ensemble_predictions

Python environment

```
Designed for Spyder / Anaconda with:
   numpy
   pandas
   scikit-learn
   openpyxl
"""


import os
import pickle
import numpy as np
import pandas as pd
from sklearn.metrics import r2_score



#
=====================================================================
==
# 1. USER SETTINGS
#
=====================================================================
==


DATA_DIR = r"C:\..."


WORKSPACE_FILES = {
   "Aurora": "Aurora_calibration_transfer_workspace.pkl",
   "ISC": "ISC_calibration_transfer_workspace.pkl",
   "JDSU": "JDSU_calibration_transfer_workspace.pkl",
}
```

```
OUTPUT_EXCEL = "reduced_top_five_ensemble_results_32responses.xlsx"


EXPECTED_N_RESPONSES = 32

EXPECTED_N_VALIDATION = 19

N_ENSEMBLE_MODELS = 5




#
===========================================================================
==

# 2. BASIC METRICS

#
===========================================================================
==


def rmse(y_true, y_pred):

   """Root mean square error."""

   y_true = np.asarray(y_true, dtype=float).ravel()

   y_pred = np.asarray(y_pred, dtype=float).ravel()

   return float(np.sqrt(np.mean((y_true - y_pred) ** 2)))




def rpd(y_true, y_pred):

   """Ratio of performance to deviation: SD(reference, ddof=1) / RMSEP."""

   y_true = np.asarray(y_true, dtype=float).ravel()

   value_rmse = rmse(y_true, y_pred)

   if value_rmse == 0:

      return np.nan

   return float(np.std(y_true, ddof=1) / value_rmse)
```

```
#
================================================================================
==
# 3. WORKSPACE UTILITIES
#
================================================================================
==


def load_workspace(path):
    """Load one workspace created by Supplementary File S1."""
    if not os.path.exists(path):
        raise FileNotFoundError(f"Workspace file not found:\n{path}")

    with open(path, "rb") as file:
        workspace = pickle.load(file)

    required_objects = {"results_df", "predictions_df"}
    missing = required_objects - set(workspace.keys())
    if missing:
        raise KeyError(
            f"The workspace is missing required objects: {sorted(missing)}"
        )

    return workspace



def prediction_method_label(results_df):
    """
    Create the method label used in predictions_df.
```

```
    In results_df, PDS is stored as:
        Method = "PDS"
        Method_detail = "window=3" (or 5, 7, 9, 11)

    In predictions_df, the corresponding label is:
        "PDS window=3"
    """
    method = results_df["Method"].astype(str).copy()
    detail = results_df["Method_detail"].astype(str)

    is_pds = method.eq("PDS")
    method.loc[is_pds] = "PDS " + detail.loc[is_pds]

    return method


#
==========================================================================
==
# 4. TOP-FIVE ENSEMBLE FOR ONE TARGET INSTRUMENT
#
==========================================================================
==

def analyse_instrument(instrument, workspace):
    """Run the reduced top-five ensemble workflow for one target instrument."""

    results = workspace["results_df"].copy()
    predictions = workspace["predictions_df"].copy()
```

```
# Convert identifier columns to ordinary strings.
result_string_cols = [
    "Response",
    "Transfer_preprocessing",
    "PLS_preprocessing",
    "Method",
    "Method_detail",
]
prediction_string_cols = [
    "ID",
    "Response",
    "Transfer_preprocessing",
    "PLS_preprocessing",
    "Method",
]

for col in result_string_cols:
    results[col] = results[col].astype(str)

for col in prediction_string_cols:
    predictions[col] = predictions[col].astype(str)

remaining_responses = sorted(results["Response"].unique())

if len(remaining_responses) != EXPECTED_N_RESPONSES:
    raise ValueError(
        f"{instrument}: {len(remaining_responses)} response variables found; "
        f"expected {EXPECTED_N_RESPONSES}."
```

```
    )

# ------------------------------------------------------------------------
# Ensemble selection uses transferred models only.
# ------------------------------------------------------------------------
transferred = results[
    results["Method"] != "Original master benchmark"
].copy()

transferred["Prediction_method"] = prediction_method_label(transferred)
transferred["Abs_bias"] = transferred["Bias"].abs()
transferred["Slope_distance"] = (transferred["Slope"] - 1.0).abs()

# Prepare prediction subsets once for efficient matching.
prediction_groups = {
    response: df.copy()
    for response, df in predictions.groupby("Response", sort=False)
}

response_rows = []
selected_rows = []
ensemble_prediction_rows = []

config_keys = [
    "Response",
    "Transfer_preprocessing",
    "PLS_preprocessing",
    "Prediction_method",
]
```

```
for response, candidate_df in transferred.groupby("Response", sort=True):

    candidate_df = candidate_df.copy()

    # --------------------------------------------------------------------
    # Composite ranking.
    # pandas rank(method="average") reproduces the original ranking logic.
    # --------------------------------------------------------------------
    candidate_df["Rank_RMSEP"] = candidate_df["RMSEP"].rank(
        method="average", ascending=True
    )
    candidate_df["Rank_R2"] = candidate_df["R2_pred"].rank(
        method="average", ascending=False
    )
    candidate_df["Rank_abs_bias"] = candidate_df["Abs_bias"].rank(
        method="average", ascending=True
    )
    candidate_df["Rank_RPD"] = candidate_df["RPD"].rank(
        method="average", ascending=False
    )
    candidate_df["Rank_slope"] = candidate_df["Slope_distance"].rank(
        method="average", ascending=True
    )

    candidate_df["Composite_rank"] = candidate_df[
        [
            "Rank_RMSEP",
            "Rank_R2",
```

```
        "Rank_abs_bias",
        "Rank_RPD",
        "Rank_slope",
    ]
].sum(axis=1)

# Stable deterministic tie-breaking after the composite rank.
top_five = candidate_df.sort_values(
    by=[
        "Composite_rank",
        "RMSEP",
        "R2_pred",
        "Abs_bias",
        "Slope_distance",
    ],
    ascending=[True, True, False, True, True],
).head(N_ENSEMBLE_MODELS).copy()

top_five["Ensemble_rank"] = np.arange(
    1, N_ENSEMBLE_MODELS + 1
)
top_five.insert(0, "Instrument", instrument)

selected_rows.append(top_five)

# --------------------------------------------------------------------
# Match the five selected configurations to sample-level predictions.
# --------------------------------------------------------------------
response_predictions = prediction_groups[response].copy()
```

```
response_predictions = response_predictions.rename(
    columns={"Method": "Prediction_method"}
)

selected_keys = top_five[config_keys].copy()

selected_predictions = response_predictions.merge(
    selected_keys,
    on=config_keys,
    how="inner",
    validate="many_to_one",
)

# Each validation sample must contain five predictions.
ensemble_predictions = (
    selected_predictions
    .groupby("ID", sort=False)
    .agg(
        Observed=("Observed", "first"),
        Ensemble_prediction=("Predicted", "mean"),
        Ensemble_prediction_SD=("Predicted", "std"),
        N_models=("Predicted", "size"),
    )
    .reset_index()
)

if len(ensemble_predictions) != EXPECTED_N_VALIDATION:
    raise ValueError(
        f"{instrument} / {response}: "
```

```
        f"{len(ensemble_predictions)} validation samples found; "
        f"expected {EXPECTED_N_VALIDATION}."
    )

if not (
    ensemble_predictions["N_models"].min() == N_ENSEMBLE_MODELS
    and ensemble_predictions["N_models"].max() == N_ENSEMBLE_MODELS
):
    raise ValueError(
        f"{instrument} / {response}: not all validation samples "
        f"contain exactly {N_ENSEMBLE_MODELS} selected predictions."
    )

y_true = ensemble_predictions["Observed"].to_numpy(dtype=float)
y_ens = ensemble_predictions[
    "Ensemble_prediction"
].to_numpy(dtype=float)

rmsep_ens = rmse(y_true, y_ens)
r2_ens = float(r2_score(y_true, y_ens))
rpd_ens = rpd(y_true, y_ens)

# Best single configuration among the selected top five.
best_single_rmsep = float(top_five["RMSEP"].min())

rmsep_change_pct = (
    (rmsep_ens - best_single_rmsep)
    / best_single_rmsep
    * 100.0
```

```
    )

    response_rows.append(
        {
            "Instrument": instrument,
            "Response": response,
            "RMSEP_ens": rmsep_ens,
            "R2_pred_ens": r2_ens,
            "RPD_ens": rpd_ens,
            "Best_single_RMSEP_top5": best_single_rmsep,
            "RMSEP_change_pct": rmsep_change_pct,
            "Improved": rmsep_change_pct < 0,
            "Within_pm5": abs(rmsep_change_pct) <= 5,
            "Worsening_gt5": rmsep_change_pct > 5,
        }
    )

    ensemble_predictions.insert(0, "Response", response)
    ensemble_predictions.insert(0, "Instrument", instrument)
    ensemble_prediction_rows.append(ensemble_predictions)

response_metrics = pd.DataFrame(response_rows)
selected_top5 = pd.concat(selected_rows, ignore_index=True)
ensemble_predictions = pd.concat(
    ensemble_prediction_rows, ignore_index=True
)

return response_metrics, selected_top5, ensemble_predictions
```

```
# ==============================================================================
# 5. TABLE 9 SUMMARY
# ==============================================================================


def make_table9_summary(response_metrics):
    """Aggregate response-level ensemble results to the manuscript Table 9."""

    rows = []

    instrument_order = ["Aurora", "ISC", "JDSU"]

    for instrument in instrument_order:
        df = response_metrics[
            response_metrics["Instrument"] == instrument
        ].copy()

        if len(df) != EXPECTED_N_RESPONSES:
            raise ValueError(
                f"{instrument}: Table 9 aggregation contains {len(df)} "
                f"responses; expected {EXPECTED_N_RESPONSES}."
            )

        rows.append(
            {
                "Instrument": instrument,
```

```
            "R2_ge_0.70": int((df["R2_pred_ens"] >= 0.70).sum()),
            "R2_ge_0.50": int((df["R2_pred_ens"] >= 0.50).sum()),
            "Improved": int(df["Improved"].sum()),
            "Within_pm5": int(df["Within_pm5"].sum()),
            "Worsening_gt5": int(df["Worsening_gt5"].sum()),
            "Mean_change_pct": df["RMSEP_change_pct"].mean(),
            "Median_change_pct": df["RMSEP_change_pct"].median(),
            "Mean_R2_pred_ens": df["R2_pred_ens"].mean(),
            "Median_R2_pred_ens": df["R2_pred_ens"].median(),
            "Mean_RPD_ens": df["RPD_ens"].mean(),
            "N_response_variables": len(df),
        }
    )

    return pd.DataFrame(rows)



# ================================================================================
# 6. MAIN WORKFLOW
# ================================================================================


def main():

    all_response_metrics = []
    all_selected_top5 = []
    all_ensemble_predictions = []
```

```
for instrument, filename in WORKSPACE_FILES.items():

    workspace_path = os.path.join(DATA_DIR, filename)

    print("\n" + "=" * 80)
    print(f"Processing {instrument}")
    print(f"Workspace: {workspace_path}")
    print("=" * 80)

    workspace = load_workspace(workspace_path)

    (
        response_metrics,
        selected_top5,
        ensemble_predictions,
    ) = analyse_instrument(instrument, workspace)

    all_response_metrics.append(response_metrics)
    all_selected_top5.append(selected_top5)
    all_ensemble_predictions.append(ensemble_predictions)

response_metrics_df = pd.concat(
    all_response_metrics, ignore_index=True
)
selected_top5_df = pd.concat(
    all_selected_top5, ignore_index=True
)
ensemble_predictions_df = pd.concat(
```

```
        all_ensemble_predictions, ignore_index=True
    )

    table9_df = make_table9_summary(response_metrics_df)

    output_path = os.path.join(DATA_DIR, OUTPUT_EXCEL)

    with pd.ExcelWriter(output_path, engine="openpyxl") as writer:
        table9_df.to_excel(
            writer, sheet_name="Table9_summary", index=False
        )
        response_metrics_df.to_excel(
            writer, sheet_name="Response_metrics", index=False
        )
        selected_top5_df.to_excel(
            writer, sheet_name="Selected_top5", index=False
        )
        ensemble_predictions_df.to_excel(
            writer, sheet_name="Ensemble_predictions", index=False
        )

    print("\nAnalysis completed.")
    print("\nCorrected Table 9:")
    print(table9_df.to_string(index=False))
    print(f"\nResults saved to:\n{output_path}")


if __name__ == "__main__":
    main()
```
