# Supplementary material for: From Benchtop to Handheld Systems: Calibration Transfer for On-Site NIR System Butter Quality Monitoring
Source: Sensors (Basel). 2026 Sep 26;26(19):6110. doi: 10.3390/s26196110 (PMC13667566; doi:10.3390/s26196110)
Supplement: Supplementary file 1 [file sensors-26-06110-s001.zip › sensors-4504687-supplementary S3.pdf]

Supplementary File S3: Python code used to generate the official Kennard–Stone sample split

```
# -*- coding: utf-8 -*-
"""
=====================

Creation of the official Kennard-Stone sample split used in the study.

PURPOSE
-------
This script generates the fixed sample allocation used throughout the
calibration-transfer analyses. The Kennard-Stone algorithm is applied ONCE to the
DS2500 (DS25) master spectra only. Target-instrument spectra are not used for sample
allocation.

The resulting split is saved as:
   kennard_stone_split_official.csv

The same file is subsequently imported by Supplementary File S1 with:
   USE_EXISTING_KS_SPLIT = True

EXPERIMENTAL DESIGN
-------------------
Total samples: 99

Sequential Kennard-Stone allocation:
   20 samples -> standardization
   19 samples -> validation
```

10 samples -> update

50 remaining samples -> calibration

INPUT

DS2500.csv

Required structure:

- one sample-identifier column named "ID";
- spectral columns whose names are numeric wavelengths.

OUTPUT

------

kennard_stone_split_official.csv

The output contains:

ID, Subset

where Subset is one of:

standardization

calibration

update

validation

NOTES

- The split is generated from the master instrument only.
- Spectral variables are standardized before Euclidean-distance calculations.
- The selection is deterministic.

```
- The output file is intended to be created once and then reused unchanged in
  all subsequent calibration-transfer analyses.

Python environment
------------------
Designed for Spyder / Anaconda with:
   numpy
   pandas
   scikit-learn
"""

import os
import numpy as np
import pandas as pd
from sklearn.preprocessing import StandardScaler


#
==============================================================================
==
# 1. USER SETTINGS
#
==============================================================================
==

DATA_DIR = r"C:\..."

MASTER_FILE = "DS2500.csv"
ID_COL = "ID"
```

```
OUTPUT_SPLIT = "kennard_stone_split_official.csv"

N_STANDARDIZATION = 20
N_VALIDATION = 19
N_UPDATE = 10
N_CALIBRATION = 50


# ============================================================================
# 2. FILE UTILITIES
# ============================================================================

def clean_column_names(df):
   """Remove leading/trailing spaces and hidden UTF-8 BOM characters."""
   df = df.copy()
   df.columns = (
      df.columns
      .astype(str)
      .str.replace("\ufeff", "", regex=False)
      .str.strip()
   )
   return df


def read_table(filepath):
   """Read CSV or Excel files and clean column names."""
```

```
    if not os.path.exists(filepath):
        raise FileNotFoundError(
            f"Input file not found:\n{filepath}\n"
            "Please check DATA_DIR and MASTER_FILE."
        )

    ext = os.path.splitext(filepath)[1].lower()

    if ext == ".csv":
        df = pd.read_csv(
            filepath,
            sep=None,
            engine="python",
            encoding="utf-8-sig"
        )
    elif ext in [".xlsx", ".xls"]:
        df = pd.read_excel(filepath)
    else:
        raise ValueError(f"Unsupported file format: {ext}")

    return clean_column_names(df)


def get_spectral_columns(df, id_col="ID"):
    """Identify wavelength columns from numeric column names."""
    spectral_cols = []

    for col in df.columns:
        if col == id_col:
```

```
            continue

        try:
            float(col)
            spectral_cols.append(col)
        except ValueError:
            continue

    return sorted(spectral_cols, key=lambda x: float(x))


#
=================================================================================
==
# 3. KENNARD-STONE ALGORITHM
#
=================================================================================
==

def kennard_stone(X, n_samples):
    """
    Deterministic Kennard-Stone selection based on Euclidean distances in
    standardized spectral space.

    The two most distant samples are selected first. Subsequent samples are
    chosen iteratively as those having the greatest minimum distance from the
    already selected set.
    """
    X = np.asarray(X, dtype=float)
```

```
if n_samples > X.shape[0]:
    raise ValueError(
        "n_samples cannot exceed the number of available samples."
    )

# Standardize wavelength variables before distance calculation.
Xs = StandardScaler().fit_transform(X)

# Pairwise Euclidean distance matrix.
diff = Xs[:, np.newaxis, :] - Xs[np.newaxis, :, :]
dist = np.sqrt(np.sum(diff ** 2, axis=2))

# Initial pair: two most distant samples.
i, j = np.unravel_index(np.argmax(dist), dist.shape)

selected = [int(i), int(j)]
remaining = sorted(
    list(set(range(X.shape[0])) - set(selected))
)

# Sequentially select the sample with the largest minimum distance
# from the already selected samples.
while len(selected) < n_samples:
    min_dist = np.min(
        dist[remaining][:, selected],
        axis=1
    )

    next_position = int(np.argmax(min_dist))
```

```
        next_idx = int(remaining[next_position])

        selected.append(next_idx)
        remaining.remove(next_idx)
        remaining = sorted(remaining)

    return (
        np.asarray(selected, dtype=int),
        np.asarray(remaining, dtype=int)
    )


#
===========================================================================
==
# 4. SEQUENTIAL SPLIT
#
===========================================================================
==

def sequential_kennard_stone_split(X_master):
    """
    Generate the official split from MASTER spectra only.

    Allocation order:
        20 standardization
        19 validation
        10 update
        50 calibration
    """
```

```
all_indices = np.arange(X_master.shape[0])

# 1. Standardization set.
std_local, rem1_local = kennard_stone(
    X_master,
    N_STANDARDIZATION
)
idx_std = all_indices[std_local]
idx_rem1 = all_indices[rem1_local]

# 2. Hold-out validation set from the remaining samples.
val_local, rem2_local = kennard_stone(
    X_master[idx_rem1, :],
    N_VALIDATION
)
idx_val = idx_rem1[val_local]
idx_rem2 = idx_rem1[rem2_local]

# 3. Update set from the remaining samples.
upd_local, rem3_local = kennard_stone(
    X_master[idx_rem2, :],
    N_UPDATE
)
idx_upd = idx_rem2[upd_local]

# 4. All remaining samples form the calibration set.
idx_cal = idx_rem2[rem3_local]

if len(idx_cal) != N_CALIBRATION:
```

```
        raise ValueError(
            f"Calibration set has {len(idx_cal)} samples, "
            f"expected {N_CALIBRATION}."
        )

    return {
        "standardization": idx_std,
        "calibration": idx_cal,
        "update": idx_upd,
        "validation": idx_val,
    }


# ==================================================================
==
# 5. OUTPUT TABLE
# ==================================================================
==

def build_split_dataframe(sample_ids, split, id_col="ID"):
    """Create the ID-to-subset assignment table."""
    split_df = pd.DataFrame({
        id_col: sample_ids,
        "Subset": "not_assigned"
    })

    split_df.loc[
        split["standardization"],
```

```
        "Subset"
    ] = "standardization"

    split_df.loc[
        split["calibration"],
        "Subset"
    ] = "calibration"

    split_df.loc[
        split["update"],
        "Subset"
    ] = "update"

    split_df.loc[
        split["validation"],
        "Subset"
    ] = "validation"

    return split_df


def validate_split_counts(split_df):
    """Verify that the final split has the expected sample counts."""
    expected_counts = {
        "standardization": N_STANDARDIZATION,
        "calibration": N_CALIBRATION,
        "update": N_UPDATE,
        "validation": N_VALIDATION,
    }
```

```
    observed_counts = (
        split_df["Subset"]
        .value_counts()
        .to_dict()
    )

    for subset_name, expected_n in expected_counts.items():
        observed_n = int(
            observed_counts.get(subset_name, 0)
        )

        if observed_n != expected_n:
            raise ValueError(
                f"Subset '{subset_name}' contains "
                f"{observed_n} samples; expected {expected_n}."
            )


#
==============================================================================
==
# 6. MAIN WORKFLOW
#
==============================================================================
==

def main():
    """Generate and save the official Kennard-Stone split."""
```

```python
master_path = os.path.join(
    DATA_DIR,
    MASTER_FILE
)

output_path = os.path.join(
    DATA_DIR,
    OUTPUT_SPLIT
)

print("\n" + "=" * 80)
print("Official Kennard-Stone split generation")
print("Input: DS2500 master spectra only")
print("=" * 80)

master_df = read_table(master_path)

if ID_COL not in master_df.columns:
    raise KeyError(
        f"Column '{ID_COL}' was not found in the master file."
    )

master_df[ID_COL] = (
    master_df[ID_COL]
    .astype(str)
    .str.strip()
)

spectral_cols = get_spectral_columns(
```

```
    master_df,
    id_col=ID_COL
)

if len(spectral_cols) == 0:
    raise ValueError(
        "No numeric wavelength columns were found."
    )

X_master = master_df[
    spectral_cols
].values.astype(float)

sample_ids = master_df[
    ID_COL
].values.astype(str)

expected_total = (
    N_STANDARDIZATION
    + N_VALIDATION
    + N_UPDATE
    + N_CALIBRATION
)

if X_master.shape[0] != expected_total:
    raise ValueError(
        f"The master dataset contains {X_master.shape[0]} samples, "
        f"but the requested split requires {expected_total}."
    )
```

```python
    print(f"Number of samples: {X_master.shape[0]}")
    print(f"Number of wavelengths: {X_master.shape[1]}")

    split = sequential_kennard_stone_split(
        X_master
    )

    split_df = build_split_dataframe(
        sample_ids,
        split,
        id_col=ID_COL
    )

    validate_split_counts(split_df)

    split_df.to_csv(
        output_path,
        index=False
    )

    print("\nOfficial split generated successfully.")
    print("\nSubset counts:")
    print(split_df["Subset"].value_counts())

    print(f"\nSaved to:\n{output_path}")


if __name__ == "__main__":
```

```
main()
```
